# A HU‐like protein is required for full virulence in *Xanthomonas campestris* pv. *campestris*

**DOI:** 10.1111/mpp.13128

**Published:** 2021-08-23

**Authors:** Qian Su, Xin‐Xin Wang, Ming Leng, Yan‐Hua Qi, Fu‐Yuan Pang, Ji‐Liang Tang, Guang‐Tao Lu

**Affiliations:** ^1^ State Key Laboratory for Conservation and Utilization of Subtropical Agro‐bioresources College of Life Science and Technology Guangxi University Nanning China

**Keywords:** complementary function, Fis‐like protein, HU‐like protein, type III secretion, virulence

## Abstract

Bacteria harbour several abundant small DNA‐binding proteins known as nucleoid‐associated proteins (NAPs) that contribute to the structure of the bacterial nucleoid as well as to gene regulation. Although the function of NAPs as global transcriptional regulators has been comprehensively studied in the model organism *Escherichia coli*, their regulatory functions in other bacteria remain relatively poorly understood. *Xanthomonas campestris* pv. *campestris* (Xcc) is a gram‐negative bacterium that causes black rot disease in almost all members of the crucifer family. In previous work, we demonstrated that a Fis homologue protein, which we named Fis‐like protein (Flp), contributes to the regulation of virulence, type III secretion, and a series of other phenotypes in Xcc. Here we have examined the role of *XC_1355*, which is predicted to encode a DNA‐binding protein belonging to the HU family herein named HU‐like protein (Hlp). We show that mutation of *XC_1355* in Xcc reduces the virulence, extracellular polysaccharide production, and cell motility, but has no effect on the production of extracellular enzymes and induction of the hypersensitive response. These data together with transcriptome analysis indicate that *hlp* is a previously uncharacterized gene involved in virulence that has partially overlapping and complementary functions with *flp* in Xcc, although the two regulators have opposite effects on the expression of genes involved in type III secretion. The findings add to our understanding of the complex regulatory pathways that act to regulate virulence in Xcc.

## Graphical abstract

A previously uncharacterized protein, Hlp, is involved in virulence and has opposite effects on the expression of type III secretion proteins compared with Flp.

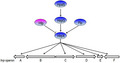

## 1 INTRODUCTION

Bacteria harbour several abundant small DNA‐binding proteins that are collectively referred to as nucleoid‐associated proteins (NAPs). These bind DNA and contribute to the structure of the bacterial nucleoid (Dillon & Dorman, 2010). Multiple types of NAP have been identified, with at least 12 distinct types in *Escherichia coli* (Azam & Ishihama, 1999; Dillon & Dorman, 2010). Several of these NAPs have been intensively studied and well characterized; these include factor for inversion stimulation (Fis), histone‐like nucleoid structuring (H‐NS), heat unstable (HU), and integration host factor (IHF). NAPs of the HU, IHF, and H‐NS families are often referred to as histone‐like proteins (HLPs), because in common with eukaryotic histones they exhibit low molecular mass, high copy number, high electrostatic charge, and strong DNA‐binding ability (Grove, 2011; Kamashev et al., 2017).

It is known that Fis influences DNA topology by directly binding and bending DNA (for review, see Dillon & Dorman, 2010). HU binds to DNA nonspecifically but with a higher affinity for nicked, gapped, and cruciform DNA structures. H‐NS is also described as binding nonspecifically to DNA but prefers intrinsically curved DNA. IHF usually binds and bends DNA strongly at specific sequences. In addition to their roles in maintaining bacterial chromosome architecture and regulating DNA transactions such as recombination and DNA replication, there is an increasing body of work describing how NAPs or their homologues also play important roles in gene regulation. For example, Fis is involved in controlling the expression of virulence factors in a number of pathogens (Duprey et al., 2014; Leng et al., 2019; Lv et al., 2018), where it regulates gene expression by modulating the level of DNA supercoiling in the cell and interacting with RNA polymerase at the position of its binding site (Dillon & Dorman, 2010). HU regulates the expression of hundreds of genes across the whole genome in *E*. *coli* by remodelling bacterial nucleoids, modulating the 3D arrangement of DNA, facilitating DNA looping in a promoter region, trapping free supercoils, indirectly altering supercoiling through DNA topoisomerases, or cooperating with transcription regulators (Berger et al., 2016; Lioy et al., 2018; Remesh et al., 2020).

NAPs in a single organism often have overlapping and complementary functions, although the underlying mechanisms are still unknown. In *E. coli*, null mutations in genes encoding each of the NAPs generally have minor consequences for the cell, showing that each individual protein is not essential for this bacterium. However, disruption of multiple NAPs causes more severe effects (Yasuzawa et al., 1992).

Although the function of NAPs as global transcriptional regulators has been comprehensively studied in the model organism *E*. *coli*, their regulatory function and associated mechanisms in other bacteria remain poorly understood.

*Xanthomonas campestris* pv. *campestris* (Xcc) is a gram‐negative bacterium that causes black rot disease in almost all members of the crucifer family (Brassicaceae), including important vegetables such as broccoli, Brussels sprouts, cabbage, cauliflower, kale, mustard, radish, and rape, and the model plant *Arabidopsis thaliana* (Vicente & Holub, 2013). This phytopathogen infects host plants via wounds or hydathodes. After infection, the bacterial cells multiply in the intercellular spaces, spread via the vascular system, and lead to the development of typical disease symptoms: vein blackening and V‐shaped chlorotic and necrotic lesions extending from leaf margins along veins (Chan & Goodwin, 1999).

Xcc encodes a number of virulence factors, such as type III secretion system (T3SS)‐dependent effectors, cyclic glucans, lipopolysaccharides, extracellular polysaccharide (EPS, also called xanthan gum), and a series of extracellular enzymes including amylase, endoglucanase, polygalacturonate lyase, and protease (Büttner & Bonas, 2010; Ryan et al., 2011). These virulence factors act in diverse ways to promote Xcc virulence. Complex regulatory networks, including quorum‐sensing pathways, multiple two‐component systems, and transcriptional regulators, act to ensure appropriate expression of these virulence factors to promote successful invasion and proliferation during pathogenesis.

Although the regulation of virulence in Xcc has been studied over the past several decades, our understanding of this complex of regulatory pathways is not complete. In particular, the role of NAPs has received comparatively little attention. In a previous work, we demonstrated that a Fis homologue protein, which we named Fis‐like protein (Flp), contributes to the regulation of virulence, expression of T3SS genes, and a series of other phenotypes in Xcc (Leng et al., 2019). Here we have examined the role of *XC_1355*, which is predicted to encode a DNA‐binding protein belonging to the HU family herein named HU‐like protein (Hlp). We show that mutation of *XC_1355* in Xcc reduces the virulence, EPS production, and cell motility, but has no effect on the production of extracellular enzymes and induction of the hypersensitive response (HR). Moreover, overexpression of *XC_1355* in the *flp* deletion mutant Δ*flp* partially restored the virulence towards wild type. These data together with transcriptome analysis indicate that *hlp* is a previously uncharacterized gene involved in virulence that has partially overlapping and complementary functions with *flp* in Xcc.

## 2 RESULTS

### 2.1 The HU family protein Hlp influences the virulence of Xcc

In our previous work we identified and characterized a Fis‐like protein (named Flp) that plays an important role in virulence and T3SS gene expression in Xcc (Leng et al., 2019). Analysis of Xcc genome sequences (da Silva et al., 2002; Qian et al., 2005; Vorhölter et al., 2008) revealed a further 12 genes that probably encode NAPs (Table 1). Homologues of some of these proteins have been implicated in the virulence of other *Xanthomonas* species. In particular, HupB is implicated in flagellar motility and virulence in *Xanthomonas citri* (Conforte et al., 2019), whereas XvrA, XvrB, and XvrC are implicated in virulence in *Xanthomonas oryzae* (Feng et al., 2009; Kametani‐Ikawa et al., 2011; Liu et al., 2016).

**TABLE 1:** Genes predicted to encode NAPs (Fis, HU, IHF, H‐NS, and HC2 families) in *Xanthomonas campestris* pv. *campestris*

Gene ID	Name	Predicted product (Qian et al., 2005)	Length (amino acids)	Domain	Accession number	Position (amino acids)	E‐value
*XC_0520*	*flp*	Fis family transcriptional regulator	90	Helix‐turn‐helix_8	PF02954	46–87	2.7e−16
*XC_1046*	*xrvC*	H‐NS family nucleoid protein	130	Histone‐like protein of HNS family (HNS)	SM00528	75–118	4.06e−10
*XC_1234*		Histone H1	156	Not found			
*XC_1355*	*hlp*	Histone‐like protein	140	Bacterial histone‐like domain	SM00411	43–139	4.00e−28
*XC_1656*	*ihfA*	Integration host factor alpha chain	99	Bacterial histone‐like domain	SM000411	3–92	7e−59
*XC_1806*	*xrvA*	H‐NS family nucleoid protein	134	Histone‐like protein of HNS family (HNS)	SM000528	79–122	1.12e−12
*XC_1860*		Histone H1	86	Bacterial histone H1‐like nucleoprotein HC2	PF07382	2–66	0.023
*XC_1925*	*ihfB*	Integration host factor β subunit	103	Bacterial histone‐like domain	SM000411	1–91	1.14e−39
*XC_3262*	*hupB*	DNA‐binding protein HU‐β	90	Bacterial histone‐like domain	SM000411	1–90	6.16e−45
*XC_3597*	*xrvB*	H‐NS family nucleoid protein	155	Histone‐like protein of HNS family	SM00528	77–122	3.15e−16
*XC_3985*		Histone H1	259	Bacterial histone H1‐like nucleoprotein HC2	PF07382	162–254	0.00067
*XC_4203*		Histone H1	83	Bacterial histone H1‐like nucleoprotein HC2	PF07382	2–82	0.0016

**FIGURE 1:**
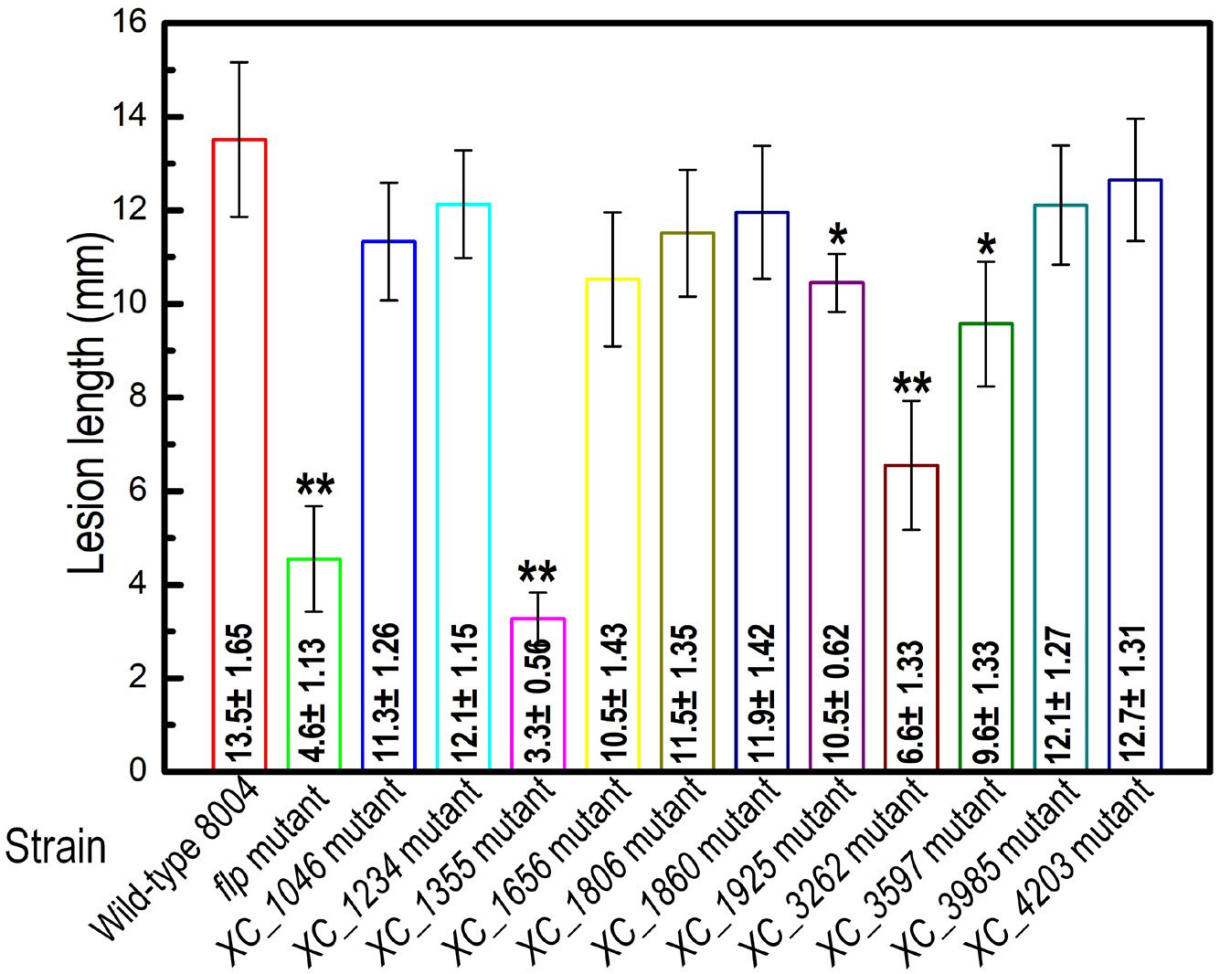
Mean lesion lengths (disease symptoms) caused by different *Xanthomonas campestris* pv. *campestris* (Xcc) mutant strains. The Xcc wild‐type strain 8004 and a series of mutant strains (Δ*flp*, 1046pk, 195B09, 1355pk, 1656pk, 1806pk, 1860nk, 247H03, 3262nk, 100B06, 147G06, 4203pk; for the corresponding genes, see Table S1) were cultured in NYG medium overnight and then adjusted to 10^7^ cfu/ml in sterile distilled water. Chinese radish (*Raphanus sativus*) was inoculated with bacterial suspensions of different Xcc strains by the leaf‐clipping method. The lesion lengths were measured at 10 days postinoculation. Values given are the mean and *SD* from 15 inoculated leaves in one experiment. Significance was determined by analysis of variance and Dunnett's post hoc test for comparison to the wild type. *p < .05; **p < .01. The experiment was repeated three times with similar results

**FIGURE 2:**
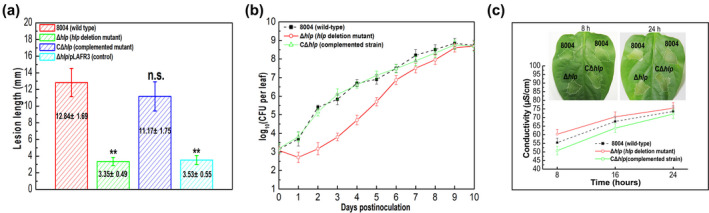
Hlp regulates virulence and in planta growth in host plants, but not the hypersensitive response (HR) in nonhost plants. (a) Lesion lengths caused by tested *Xanthomonas campestris* pv. *campestris* (Xcc) strains. The Xcc wild‐type strain 8004, the *hlp* deletion mutant Δ*hlp*, the complemented strain CΔ*hlp*, and control strain Δ*hlp*/pLAFR3 were cultured in NYG medium overnight and then adjusted to 10^7^ cfu/ml in sterile distilled water. Xcc strains were inoculated into Chinese radish by the leaf‐clipping method. Lesion lengths were scored 10 days postinoculation. Data are shown as the mean ± *SD* from 15 inoculated leaves in one experiment. Significance was determined by analysis of variance and Dunnett's post hoc test for comparison to the wild type. **p < .01; n.s., not significant. The experiment was repeated three times with similar results. (b) Growth of Xcc strains within host plants. The Xcc wild‐type strain 8004, the *hlp* deletion mutant Δ*hlp*, and complemented strains CΔ*hlp* were cultured in NYG medium overnight and then adjusted to 10^7^ cfu/ml in sterile distilled water. Xcc strains were inoculated onto radish leaves using the leaf‐clipping method. Five leaves were collected from every group of clipped leaves daily and homogenized in sterile water. The homogenates were diluted and plated on NYG plates. Bacterial cfu were counted after incubation for 3 days. Data are shown as the mean and *SD* from three replicates. (c) HR induction by Xcc strains in nonhost plants. Bacterial cells from overnight culture were resuspended in 10 mM sodium phosphate buffer to a density of 10^7^ cfu/ml. Approximately 5 μl bacterial resuspension was infiltrated into pepper (*Capsicum annuum* ‘ECW‐10R’) leaf mesophyll tissue with a blunt‐end plastic syringe. HR symptoms were recorded at 8 and 24 hr postinoculation (hpi) (top). There was electrolyte leakage from pepper leaves inoculated with Xcc strains (bottom). The conductivity of the infiltrated spots was measured by a DDS‐307A conductometer at 8, 16, and 24 hpi, with four 0.4‐cm^2^ leaf discs collected from the infiltrated area for each sample. Three samples were taken for each measurement in each experiment. Data are shown as the mean ± *SD* of three replicates from a representative experiment, and similar results were obtained in two other independent experiments

As a first step to explore the roles of these other NAPs in Xcc, we screened a series of insertional mutants that were constructed using a suicide vector pK18*mob* (Windgassen et al., 2000) or transposon Tn*5* *gusA*5 strategy (Table S1). Virulence assays in the host plant Chinese radish using a leaf‐clipping method showed that besides *flp* and *hupB* (Leng et al., 2019; Zhang et al., 2020), mutation of *XC_1355*, which is predicted to encode a DNA‐binding protein belonging to the HU family, also caused a reduction of disease symptoms when compared with the wild‐type Xcc strain (Figure 1). XC_1355 was named HU‐like protein (Hlp). In order to explore the detailed function of *hlp*, an in‐frame deletion mutant of *hlp* was constructed by using the suicide vector pK18*mobsacB*; the derived mutant strain was designated Δ*hlp*. Simultaneously, a complemented strain was constructed by introducing a recombinant pLAFR3 plasmid carrying the *hlp* coding sequence into Δ*hlp*. The resulting complemented strain was named CΔ*hlp* (Table S1). Virulence assays revealed that the Δ*hlp* mutant caused markedly reduced disease symptoms (Figure S1) compared with the wild type: the mean lesion length caused by Δ*hlp* was significantly shorter than that caused by the wild‐type strain 8004 (Figure 2a). Furthermore, CΔ*hlp* produced similar virulence symptoms to the wild type. These data confirmed the findings reported above that *hlp* is important for the virulence of Xcc.

To get more insight into the regulatory effects of Hlp on virulence in Xcc, the effects of Hlp on the proliferation of the pathogen in host tissues were assessed. To do this, the bacterial cell numbers of the wild‐type strain, the Δ*hlp* mutant, and the CΔ*hlp* complemented strain in infected Chinese radish leaves were determined on each day for 10 days after inoculation. For the Δ*hlp* mutant, the number of bacterial cells recovered from the infected leaves was 10‐ to 100‐fold less compared to the wild‐type strain up to 5 days postinoculation (Figure 2b). After 5 days the differences in bacterial numbers were smaller. The complemented strain CΔ*hlp* showed similar cell numbers to the wild type (Figure 2b). These data indicated that loss of Hlp has the strongest influence in the early invasive stage of the infection, where it reduces bacterial fitness (Figure 2b).

The growth characteristics of the Δ*hlp* mutant and the wild‐type strain in the minimal medium XVM2 were also compared. Results revealed that the Δ*hlp* strain displayed similar growth properties to the wild‐type strain in all growth phases (Figure S2). These findings indicated that the impact of mutation of *hlp* on growth in planta was not due to a general growth defect.

The *hlp* gene is predicted to encode a protein of 140 amino acids. Analysis with the Simple Modular Architecture Research Tool (SMART) program (http://smart.embl‐heidelberg.de) indicated that Hlp has (a) a domain characteristic of bacterial DNA‐binding proteins (PF00216, 5.7 × 10^−15^) at its C‐terminus (residues 43–139), (b) 42.9% (52.9%) identity (similarity) with the HU family DNA‐binding protein (accession: WP_160456856, HU_Bst_ counterpart) from *E*. *coli* (Vis et al., 1995), and (c) 16.4% (27.1%) identity (similarity) with HU_Bst_ (accession: AAA22532) from *Geobacillus stearothermophilus* (Padas et al., 1992) (Figure S3).

### 2.2 Hlp is not required for HR in nonhost plants

Our finding that Hlp is required for full virulence and in planta growth of Xcc in host plants prompted us to examine the influence of Hlp on the ability to induce HR in nonhost plants. For these experiments, the Δ*hlp* mutant was infiltrated at a cell concentration of 10^7^ cfu/ml into the leaves of the pepper cultivar ECW‐10R. The results showed that this mutant elicited similar HR symptoms to those seen with the wild‐type strain (Figure 2c), suggesting that Hlp has no obvious impact on HR induction in Xcc. The effect of Hlp on Xcc HR induction was further substantiated using the electrolyte leakage assay. The Δ*hlp* mutant induced very similar levels of electrolyte leakage to the wild type at all time points tested (Figure 2c).

### 2.3 Hlp influences extracellular polysaccharide production, cell motility, and stress tolerance, but not extracellular enzyme production

To explore the role of Hlp in the pathogenesis of Xcc, we conducted a series of basic phenotypic tests to examine the influence that mutation of *hlp* might have on the production of EPS, the production of extracellular enzymes, cell motility, and the tolerance to stress and antimicrobials.

**FIGURE 3:**
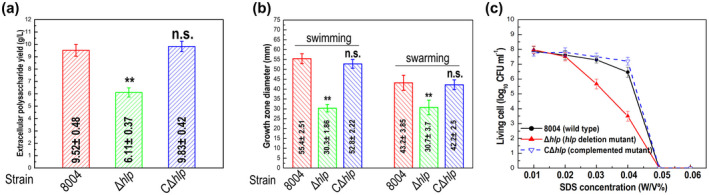
Hlp regulates a series of phenotypes in *Xanthomonas campestris* pv. *campestris* (Xcc). (a) Extracellular polysaccharide (EPS) yield of tested Xcc strains. Xcc strains were cultured in NY medium containing 2% glucose for 3 days before EPS was extracted and quantified. Data are shown as the mean ± *SD* of three replicates from a representative experiment. Significance was determined by analysis of variance (ANOVA) and Dunnett's post hoc test for comparison to the wild type. **p < .01; n.s., not significant. The experiment was repeated three times with similar results. (b) Motility of tested Xcc strains. Two microlitres of culture suspension (10^9^ cfu/ml) of Xcc strains was stabbed into “swim” (0.28% agar) medium and incubated for 4 days at 28 °C or inoculated onto “swarm” (0.6% agar) plates and incubated for 3 days at 28 °C. Colony diameters were measured. Data are shown as the mean ± *SD* of 10 measurements from a representative experiment. Significance was determined by ANOVA and Dunnett's post hoc test for comparison to the wild type. **p < .01; n.s., not significant. The experiment was repeated three times with similar results. (c) Sodium dodecyl sulphate (SDS) tolerance of tested Xcc strains. Survival experiments were performed by subculturing strains overnight on fresh NYG agar plates supplemented with different SDS concentrations. The surviving bacterial colonies on the plates were counted after incubation for 3 days

The results showed that the mutant strain Δ*hlp* produced slightly less EPS (Figure 3a) and had decreased motility (swimming and swarming, tested on 0.28% (wt/vol) agar plates and 0.6% (wt/vol) agar plates, respectively; Figure 3b). EPS production and motility of the CΔ*hlp* strain were similar to wild‐type values under the conditions tested (Figure 3a,b).

The ability of the wild‐type strain, the Δ*hlp* mutant, and the CΔ*hlp* complemented strain to tolerate environmental stresses was assessed by using growth assays.

The growth of bacterial strains on agar plates supplemented with different concentrations of environmental stressors was examined. These stressors were sodium dodecyl sulphate (SDS), hyperosmosis (NaCl), hydrogen peroxide (H_2_O_2_), phenol, and heavy metal salts CuSO_4_, CdCl_2_, and ZnSO_4_. These experiments demonstrated that compared to the wild type, the Δ*hlp* mutant showed reduced survival only in the presence of SDS, but not NaCl, H_2_O_2_, phenol, or heavy metal salts (Figures 3c and S4). Additionally, the tolerance to SDS was restored to wild‐type levels in the CΔ*hlp* complemented strain (Figure 3c).

Comparison of the production of extracellular enzymes (endoglucanase and amylase) showed no differences between the wild‐type strain, the Δ*hlp* mutant, and the CΔ*hlp* complemented strain (Figure S5).

### 2.4 Functional redundancy between Hlp and Flp

**FIGURE 4:**
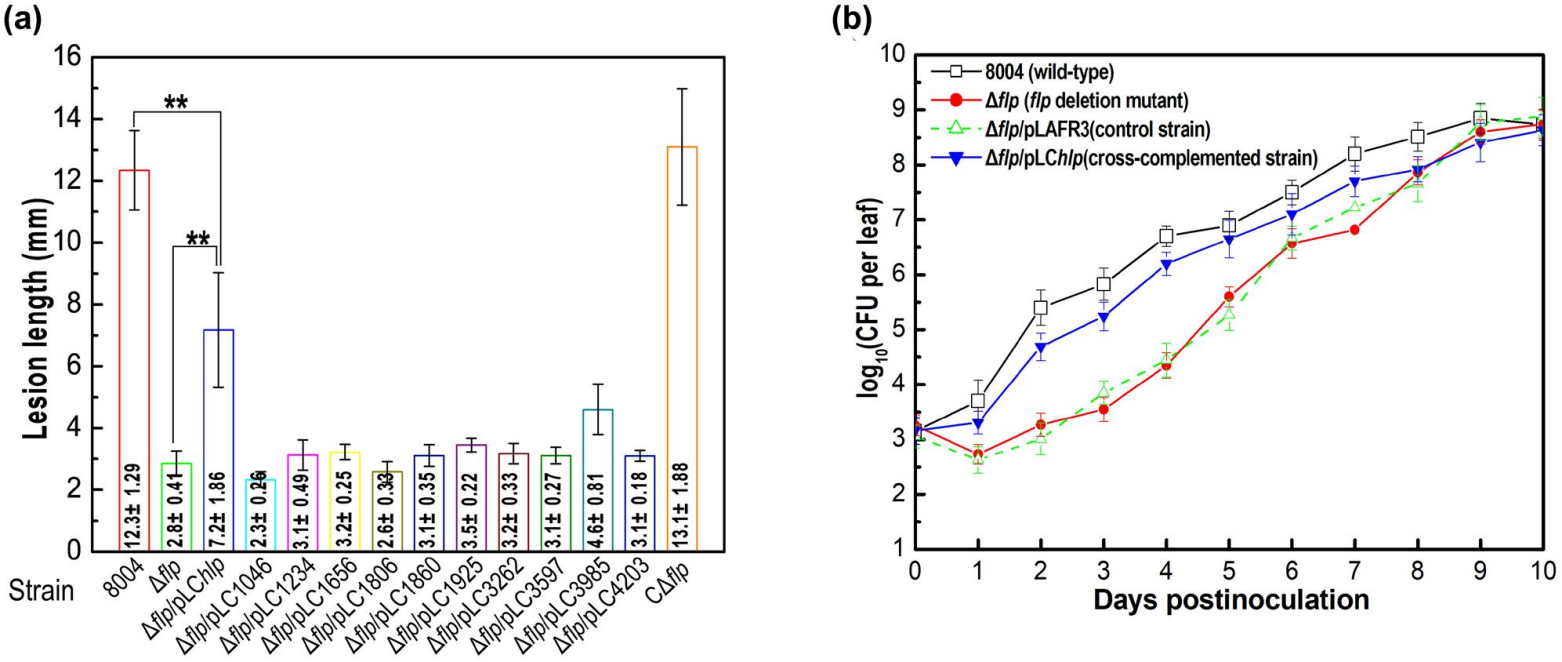
Overexpression of the *hlp* gene in the Δ*flp* mutant partially restores its virulence and growth in host plants. (a) Mean lesion lengths (disease symptoms) caused by different *Xanthomonas campestris* pv. *campestris* (Xcc) strains. The Xcc wild‐type strain 8004, the *flp* deletion mutant Δ*flp*, the complemented strain CΔ*flp*, and a series of cross‐complemented strains (Δ*flp*/pLC*hlp*, Δ*flp*/XC_1046, Δ*flp*/pLC1234, Δ*flp*/pLC1656, Δ*flp*/pLC1806, Δ*flp*/pLC1860, Δ*flp*/pLC1925, Δ*flp*/pLC3262, Δ*flp*/pLC3597, Δ*flp*/pLC3985, and Δ*flp*/pLC4203) were cultured in NYG medium overnight and then adjusted to 10^7^ cfu/ml in sterile distilled water. Chinese radish (*Raphanus sativus*) was inoculated with bacterial suspensions of different Xcc strains by the leaf‐clipping method. Lesion lengths were measured at 10 days postinoculation. Values given are the mean ± *SD* from 15 inoculated leaves in one experiment. Analysis of variance and Dunnett's post hoc test were used to identify significant differences (*p* < .01), which are indicated with asterisks. The experiment was repeated three times with similar results. (b) Growth of Xcc strains within host plants. The Xcc wild‐type strain 8004, the *flp* deletion mutant Δ*flp*, the cross‐complemented strain Δ*flp*/pLC*hlp* (Δ*flp* overexpressing *hlp*), and control strain Δ*flp*/pLAFR3 (Δ*flp* harbouring the empty plasmid pLAFR3) were cultured in NYG medium overnight and then adjusted to 10^7^ cfu/ml in sterile distilled water. Xcc strains were inoculated onto radish leaves using the leaf‐clipping method. Five leaves were collected from every group of clipped leaves daily and homogenized in sterile water. The homogenates were diluted and plated on NYG plates. Bacterial cfu were counted after incubation for 3 days. Data are shown as the mean and *SD* from three replicates

To examine any functional redundancies between Flp and Hlp (which both regulate virulence), the phenotypic effects of expression of *hlp* in the deletion mutant Δ*flp* was tested. To do this, a construct comprising the vector pLAFR3 carrying the *hlp* gene was transferred into deletion mutant Δ*flp* (Table S1). As a first step, this strain was tested for virulence in the host Chinese radish using a leaf‐clipping assay. Results revealed that the expression of *hlp* partially restored the virulence of the Δ*flp* mutant: the Δ*flp*/pLC*hlp* strain produced more severe disease symptoms with a mean lesion length of 7.2 mm than the Δ*flp* mutant with a mean lesion length of 2.8 mm, but less severe disease symptoms compared to the wild type with a mean lesion length of 12.3 mm (Figures 4a and S6).

The virulence and in planta growth of the Δ*flp* mutant, the cross‐complemented strain Δ*flp*/pLC*hlp*, and the control strain Δ*flp*/pLAFR3 in infected leaves were also estimated. Consistent with our previous work (Leng et al., 2019), the Δ*flp* mutant and the control strain Δ*flp*/pLAFR3 had lower population numbers in planta compared to the wild type in the first 5 days after inoculation (Figure 4b). Interestingly, the population size of the cross‐complemented strain Δ*flp*/pLC*hlp* was near to that of the wild type during the same period, indicating that *in trans* expression of the *hlp* gene in the Δ*flp* mutant restored in planta fitness in the early, invasive stage.

These experiments were extended to examine the effects of expression of other NAPs on the virulence of the Δ*flp* mutant (Table S1). However, none of these strains showed a change in virulence compared to the Δ*flp* mutant (Figure 4a).

**FIGURE 5:**
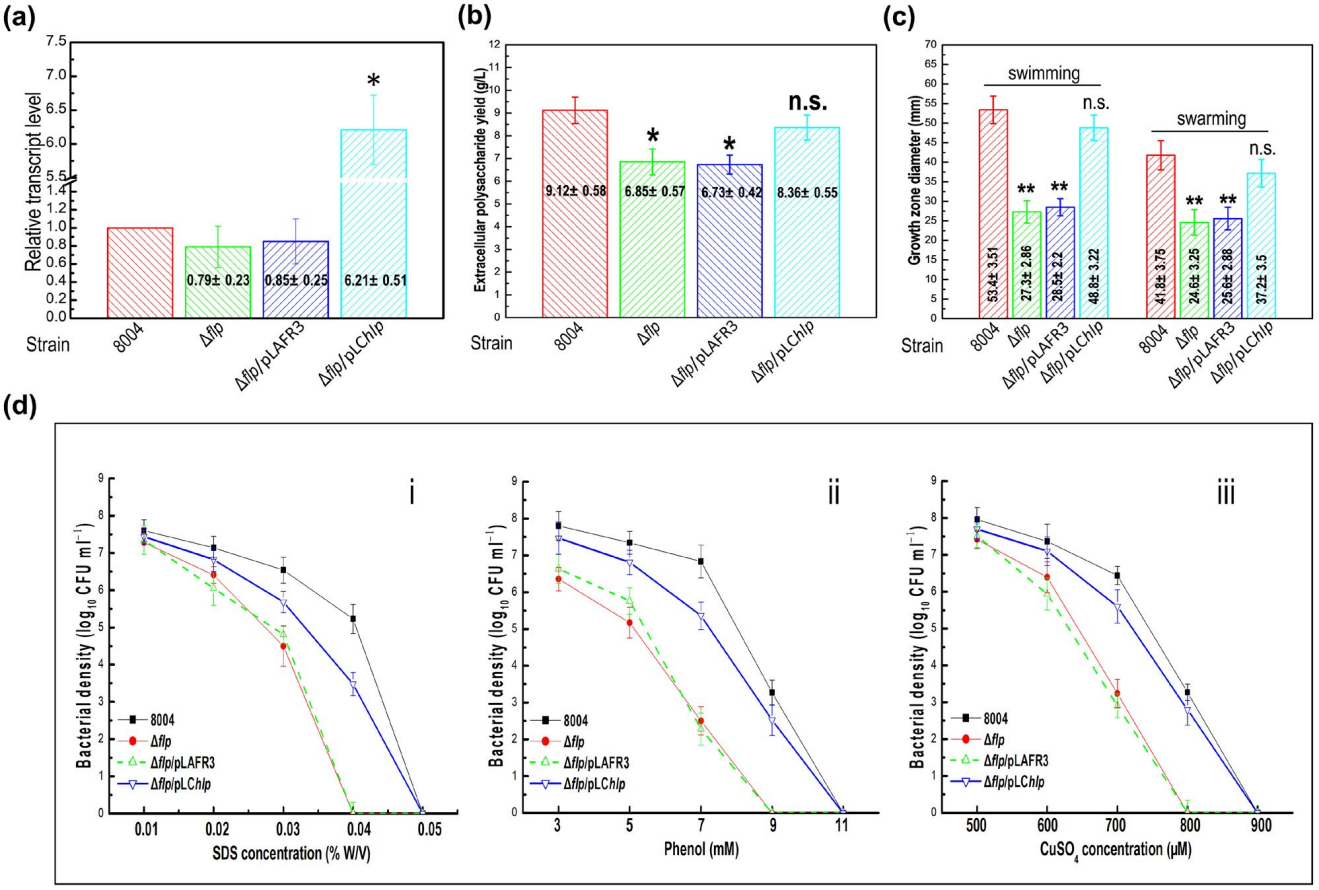
Overexpression of *hlp* in the Δ*flp* mutant restores its extracellular polysaccharide (EPS) production, cell motility, and stress tolerances. (a) Quantitative reverse transcription‐PCR assay to examine the transcription levels of *hlp* in *Xanthomonas campestris* pv. *campestris* (Xcc) strains. RNA was isolated from cultures of Xcc strains grown in XVM2 medium for 24 hr. The relative mRNA levels were calculated with respect to the level of the corresponding transcript in the wild‐type strain 8004. Values given are the means ± *SD* of triplicate measurements from a representative experiment; genes with a twofold change in expression in the mutants compared to the wild type were considered differentially expressed (*, significant). Similar results were obtained in two other independent experiments. (b) EPS yield of Xcc strains cultured in NY medium containing 2% glucose for 3 days. Data are shown as the mean ± *SD* of three replicates from a representative experiment. Significance was determined by analysis of variance (ANOVA) and Dunnett's post hoc test for comparison to the wild type. **p < .01; n.s., not significant. Similar results were obtained in two other independent experiments. (c) Measurements of colony diameters of Xcc strains in “swim” (0.28% agar) medium and on “swarm” (0.6% agar) medium after 4 and 3 days of incubation at 28 °C, respectively. Data are shown as the mean ± *SD* of 10 measurements in a representative experiment. Significance was determined by ANOVA and Dunnett's post hoc test for comparison to the wild type. **p < .01; n.s., not significant. Similar results were obtained in two other independent experiments. (d) Stress tolerance test of Xcc strains on fresh NYG agar plates supplemented with different concentrations of sodium dodecyl sulphate (SDS) (i), phenol (ii), and CuSO_4_ (iii). Values given are the means ± *SD* of triplicate measurements from a representative experiment. Similar results were obtained in two other independent experiments

In parallel, the expression of *hlp* in the cross‐complemented strain Δ*flp*/pLC*hlp* was evaluated using quantitative reverse transcription‐PCR (RT‐qPCR). Results revealed that the transcription level of *hlp* was obviously elevated in Δ*flp*/pLC*hlp* compared to that in the Δ*flp* mutant or in the wild type (Figure 5a).

Previous work revealed that Flp positively regulates EPS production, cell motility, and tolerance to certain environmental stresses (Leng et al., 2019). To further investigate any potential regulatory overlap between Hlp and Flp, we compared Xcc wild‐type strain 8004, the *flp* mutant strain Δ*flp*, and the cross‐complemented strain Δ *flp*/pLC*hlp* for EPS production, cell motility, and stress tolerance. The results showed that overexpression of *hlp* in the Δ*flp* mutant restored these phenotypes almost to wild‐type values (Figure 5). The effects of *flp* overexpression in the Δ*hlp* mutant were also estimated. No differences were seen on the tested phenotypes such as virulence, EPS production, and cell motility between the Δ*flp* mutant and the cross‐complemented strain Δ *hlp*/pLC*flp* (Figure S7).

### 2.5 Hlp controls the expression of genes involved in virulence and various adaptation processes in Xcc

To gain a better understanding of the regulatory role of Hlp in Xcc, a set of global gene expression profiles was generated using RNA sequencing (RNA‐seq). Here, the Δ*hlp* mutant and wild‐type strain 8004 were grown to the mid‐exponential phase (OD_600_ = 0.6) in XVM2, a medium that mimics more closely the nutrition environment of the plant and induces the expression of a series of virulence‐related genes (Astua‐Monge et al., 2005). The cells were collected, and total RNA was extracted from two independent biological replicates. Following library construction and sequencing, the generated data were analysed to assess differential gene expression.

**FIGURE 6:**
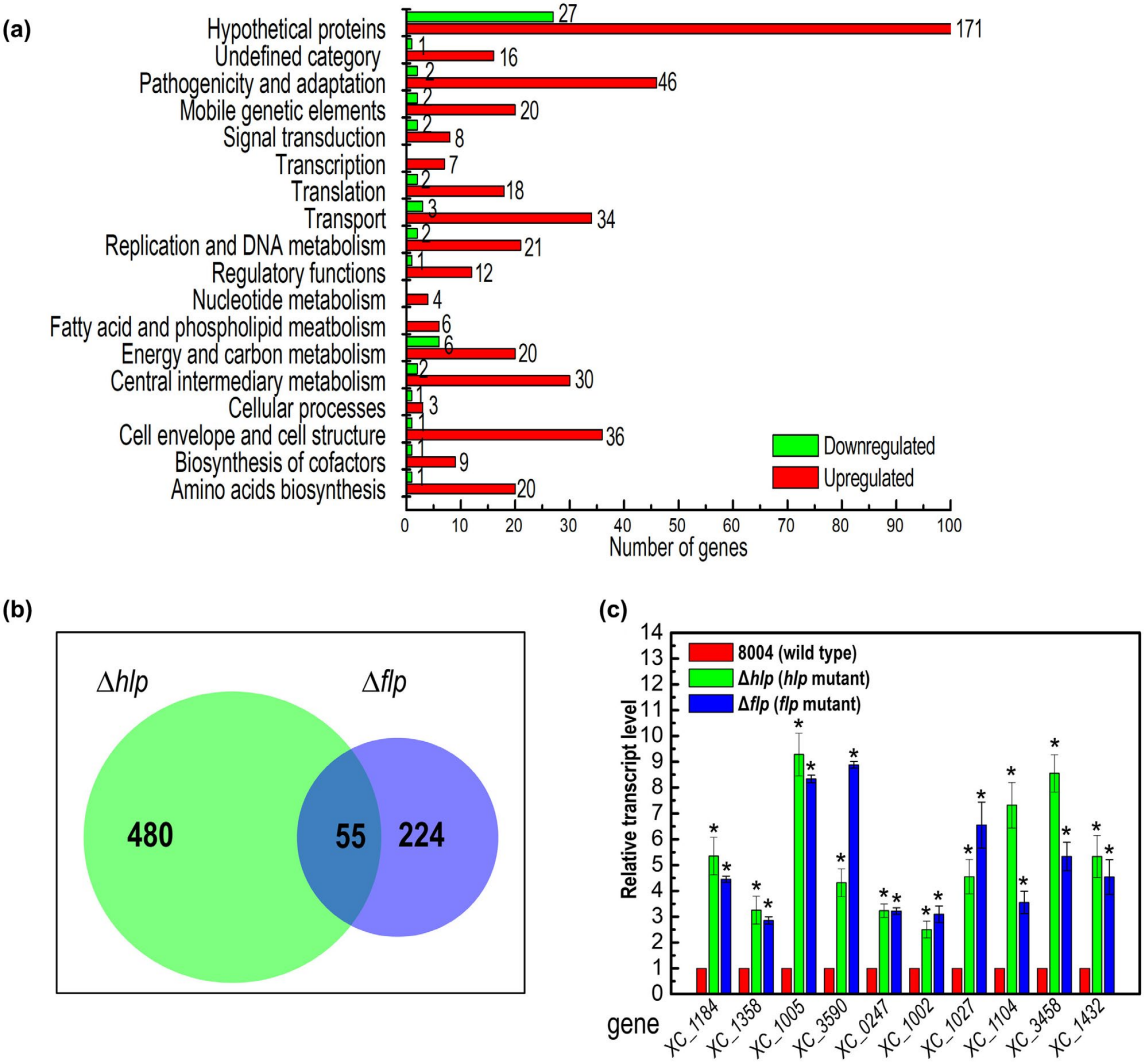
Regulatory overlap between Hlp and Flp. (a) Functional categories of differentially expressed genes in the Δ*hlp* mutant. The transcriptome of *Xanthomonas campestris* pv. *campestris* (Xcc) strains cultured in XVM2 medium was investigated by RNA‐seq, and 535 genes were found to be differentially expressed (|log_2_(fold change)| ≥ 1) in the Δ*hlp* mutant (Table S2). These genes were broadly categorized according to their biological function (He et al., 2007; Qian et al., 2005). (b) Comparison of gene expression changes in the Δ*hlp* and Δ*flp* mutants. Venn diagrams showing the overlap of genes (Table S4) whose expression is up‐regulated and down‐regulated in Δ*hlp* and Δ*flp* backgrounds. (c) Quantitative reverse transcription‐PCR assays of the expression level of several genes convergently regulated by Hlp and Flp in Xcc strains. RNA was isolated from cultures of Xcc wild‐type strain 8004, the Δ*hlp* mutant, and the Δ*flp* mutant grown in XVM2 medium for 24 hr. Relative gene expression with respect to the corresponding transcript levels in the wild‐type strain 8004 was calculated. Values given are the means ± *SD* of triplicate measurements from a representative experiment. Genes were considered to be differentially expressed if |log_2_(fold change)| ≥ 1 compared to the wild type (*, significant). Similar results were obtained in two other independent experiments

Of the 4,273 annotated genes from the genome of Xcc strain 8004, 535 genes were differentially expressed (|log_2_(fold change) ≥ 1), with 481 genes up‐regulated and 54 genes down‐regulated according to the transcriptome data (Figure 6a, Table S2), implying that Hlp mainly acts as a repressor of gene transcription in Xcc. To verify the transcriptome data, 25 differentially expressed genes (DEGs) were randomly selected and semiquantitative RT‐PCR was performed to examine the relative expression levels of these genes. All selected genes showed expression changes that were comparable with the transcriptome data (Table S3).

Functional clustering analysis was conducted according to the annotation of the Xcc strain 8004 genome (He et al., 2007; Qian et al., 2005), where the majority of the 535 genes regulated by Hlp were assigned to functional categories that are based on clusters of orthologous genes. A total of 215 genes were predicted to encode hypothetical proteins or have not been given a functional category to date (Figure 6a, Table S2). The most dominant functional category was “pathogenicity and adaption” (48 genes). Additionally, 37 genes were assigned to “transport”, 37 to “cell envelope and cell structure”, 32 to “central intermediary metabolism”, and 26 to “energy and carbon metabolism”. Notably, the expression of eight T3SS‐related genes, *XC_2994* (*xopP*), *XC_3001* (*hpa2*), *XC_3010* (*hrpB2*), *XC_3012* (*hrcU*), *XC_3016* (*hrcR*), *XC_3024* (*xopF1*), *XC_3025* (*hrpF*), and *XC_3177* (*xopQ*), was increased in the Hlp mutant compared to the wild type, implying that Hlp has effects on the T3SS.

### 2.6 Hlp and Flp convergently regulate genes involved in different phenotypes

The influence of Flp on the Xcc transcriptome has been characterized in our previous work (Leng et al., 2019). Here we compared the general overlap of genes controlled by both Flp and Hlp. In total, 55 genes were found to be regulated by both Flp and Hlp (Figure 6b, Table S4). Interestingly, 42 genes were convergently regulated by Hlp and Flp. Most of these are related to virulence and fitness, such as genes involved in cell motility (*XC_1184*, *XC_1187*, *XC_1358*, *XC_1359*) or energy or carbon mechanism (*XC_0247*, *XC_1002*, *XC_1218*, *XC_1642*, *XC_3167*) or encoding extracellular enzymes (*XC_1005*, *XC_3590*, *XC_3591*), type IV secretion system proteins (*XC_1027*), ion transporters (*XC_1104*, *XC_3458*, *XC_3459*, *XC_3463*), or drug resistance proteins (*XC_1432*, *XC_4200*). The convergent regulation of particular genes by Hlp and Flp was then verified using RT‐qPCR. RT‐qPCR analysis of 10 convergently regulated genes (*XC_1184*, *XC_1358*, *XC_1005*, *XC_3590*, *XC_0247*, *XC_1002*, *XC_1027*, *XC_1104*, *XC_3458*, *XC_1432*) in the wild type, the Δ*hlp* mutant, and the Δ*flp* mutant cultured in XVM2 medium was performed (Figure 6c). Results demonstrated that the expression of these selected genes was consistent with the data from the transcriptome analyses (Figure 6c).

### 2.7 Hlp and Flp divergently regulate the type III secretion system in Xcc

**FIGURE 7:**
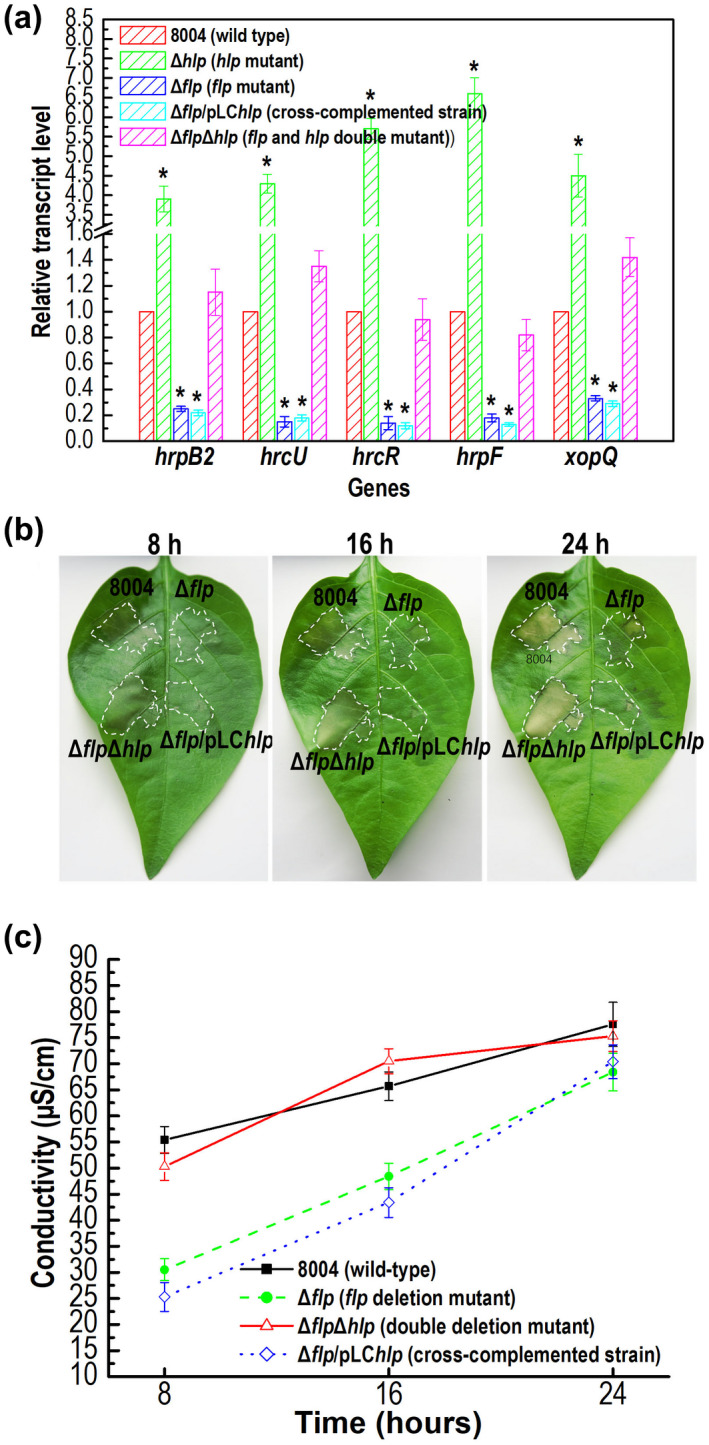
Mutation of *hlp* in the Δ*flp* mutant background restores the expression of type III secretion system (T3SS) genes. (a) The expression levels of *hrp* and type III effector (T3E) genes in *Xanthomonas campestris* pv. *campestris* (Xcc) wild‐type strain 8004, the Δ*flp* mutant, and the Δ*flp*Δ*hlp* double mutant as measured by quantitative reverse transcription‐PCR. RNA was isolated from cultures of Xcc strains grown in XVM2 medium for 24 hr. Relative gene expression with respect to the corresponding wild‐type levels were calculated. Values given are the means ± *SD* of triplicate measurements from a representative experiment. Genes were considered to be differentially expressed if |log_2_(fold change)| ≥ 1 compared to the wild type (*, significant). Similar results were obtained in two other independent experiments. (b) Hypersensitive response (HR) symptoms observed after infiltration. The Xcc wild‐type strain 8004, the Δ*flp*Δ*hlp* double mutant, the Δ*flp* mutant, and the Δ*hlp* mutant were infiltrated into pepper leaves. Three replications were performed in each experiment, and each experiment was repeated three times. The results presented are from a representative experiment, and similar results were obtained in all other independent experiments. (c) Electrolyte leakage from pepper leaves inoculated with Xcc strains. For each sample, four 0.4‐cm^2^ leaf discs were collected from the bacteria‐infiltrated area and incubated in 5 ml distilled water. Conductivity was measured with a DDS‐307A conductivity meter. Three samples were taken for each measurement in each experiment. Results presented are the mean ± *SD* of three replicates from a representative experiment, and similar results were obtained in two other independent experiments

The gene transcription profile data revealed that the expression of T3SS‐related genes was increased in the Hlp mutant but down‐regulated in the Δ*flp* mutant compared to the wild type (Tables S2 and S4). To confirm the apparent divergent impact Hlp and Flp had on the expression of T3SS, we compared the transcript levels of several *hrp* genes (*hrpB2*, *hrcU*, *hrcR*, and *hrpF*) and a T3SS effector (T3E) gene (*xopQ*) between wild type, Δ*hlp*, and Δ*flp* cultured in XVM2 medium by RT‐qPCR analysis. The results demonstrated that the expression of these selected genes was consistent with the data from the transcriptome analyses (Figure 7a).

To further investigate the interplay between Hlp and Flp in *hrp* gene regulation, we examined the expression level of several *hrp* and T3E genes in the double mutant Δ*hlp*Δ*flp* and the cross‐complemented strain Δ*flp*/pLC*hlp* by RT‐qPCR analysis. The results showed that the transcript levels of all the tested *hrp* and T3E genes in the double mutant strain Δ*hlp*Δ*flp* were at approximately wild‐type levels, while the expression levels in the cross‐complementation strain Δ*flp*/pLC*hlp* were similar to those in the Δ*flp* mutant (Figure 7a).

The Δ*hlp*Δ*flp* and Δ*flp*/pLC*hlp* strains were also examined for their ability to induce HR in nonhost plants. Bacterial suspensions at a cell concentration of 10^7^ cfu/ml were inoculated into the leaves of the pepper cultivar ECW‐10R. Consistent with the results from the RT‐qPCR analysis, the double mutant Δ*flp*Δ*hl*p induced similar levels of HR and electrolyte leakage to the wild type, while the cross‐complemented strain Δ*flp*/pLCΔ*hlp* induced a very similar HR to the Δ*flp* mutant (Figure 7b,c). Thus, although mutation of *hlp* in the wild‐type background showed no obvious effect on the ability to induce HR (Figure 2c), in the Δ*flp* mutant background it acts to restore HR induction. Taken together, these observations indicate that Hlp negatively regulates the expression of the T3SS in Xcc, whereas Flp has a positive effect.

## 3 DISCUSSION

The miscellaneous interactions between NAPs and DNA and the dynamic nucleoid architecture lead to global regulation of bacterial gene expression affecting a variety of cellular processes (Kisner & Kuwada, 2020). However, the roles of many NAPs and their functional relationships remain obscure. This is compounded by the lack of work being conducted beyond model organisms like *E. coli* and *Bacillus* species. In the present study, we used various genetic, phenotypic, and genomic methods to understand the role of *XC_1355*, which is predicted to encode a DNA‐binding protein belonging to the HU family herein named Hlp. Mutation of *XC_1355* in Xcc influences virulence, EPS production, and cell motility. While transcriptome analysis indicated that *hlp* is involved in virulence gene regulation and has partly overlapping and complementary functions with *flp*, the two regulators have opposite effects on the expression of type III secretion in Xcc. These results suggest that Hlp can regulate virulence gene expression and indicate a novel mechanism of differential regulation of T3SS gene expression to another NAP protein. Bacterial HU is a small histone‐like protein that resembles the eukaryotic histone H2B. It binds DNA as a homotypic dimer. In some organisms, such as *E*. *coli*, HU consists of two different monomers, αHU and βHU. However, in certain organisms HU has identical monomers; the HU_Bst_ protein from *Bacillus* *stearothermophilus* consists of two identical monomers of 90 amino acid residues. Extensive studies have shown that HU is essential for bacterial nucleoid condensation and remodelling that relates to global gene expression in *E. coli* (Dillon & Dorman, 2010; Meyer et al., 2018; Remesh et al., 2020), and HU proteins have been implicated in virulence and adaptation to environmental conditions in several mammalian pathogens (Hołówka et al., 2018; Sakatos et al., 2018). Nevertheless, there are relatively few reports on the role of HU proteins in plant pathogens.

Previous work has shown that a 90‐amino‐acid HU‐like protein (named HupB in *Xanthomonas citri* subsp. *citri* and HU_Xcc_ in Xcc) has been reported to regulate virulence in *Xanthomonas* spp. (Conforte et al., 2019; Zhang et al., 2020). Here deletion of HU_Xcc_ (XC_3262) in the Xcc wild‐type strain 8004 reduced disease symptoms on the host plant Chinese radish using the leaf‐clipping method (Figure 1). This is similar to the previous findings that the insertion inactivation mutant of HU_Xcc_ exhibited partially weakened virulence on the host cabbage by the infiltration method (Zhang et al., 2020). In the current study we show that a previously uncharacterized *hlp* gene from Xcc encodes a 140‐amino‐acid protein that contains a HU family DNA‐binding domain. When compared to the *E*. *coli* HU proteins, Hlp displays higher sequence similarity with the HU_Bst_ homologue protein (accession WP_160456856) (Vis et al., 1995) than with the well‐characterized αHU (25%) and βHU (26.4%). Unlike αHU and βHU in *E. coli*, the functions of HU_Bst_ in *E. coli* and its homologues in other bacterial species are poorly understood, as they have not yet been well characterized. Additionally, no such protein has been characterized in Xcc or other bacteria from the *Xanthomonas* genus. Hlp is identical in all three sequenced Xcc strains (8004, ATCC33913, and B100). Similar changes in phenotype were also observed when we mutated the *hlp* gene in the Xcc strain ATCC33913 (data not shown).

Global transcriptomics studies using RNA‐seq demonstrated that Hlp primarily acts as a repressor of gene expression under our tested conditions. Among 535 DEGs, 481 genes were up‐regulated in the *hlp* mutant. These regulated genes were involved in a range of biological functions, including virulence, membrane transport, multidrug resistance, amino acid biosynthesis (sulphur metabolism, *cys*), the T3SS, the type IV pilus, and signal transduction.

Our work has also addressed the regulatory interplay between Hlp and Flp, a Fis homologue in Xcc that we have previously shown to be a global regulator implicated in virulence and HR induction (Leng et al., 2019). The ability of *hlp* to partially restore the virulence of the Δ*flp* mutant in host plants suggest that some overlapping and complementary functions exist between Hlp and Flp. Similar to Fis in other bacteria (Duprey et al., 2014), Flp can be either a repressor or an activator of transcription in Xcc. Of the 4,273 annotated genes from the genome of Xcc strain 8004, 279 genes were found to be regulated by Flp. Among them, 121 genes were up‐regulated and 158 genes were down‐regulated (Leng et al., 2019). Our findings establish an overlap between the genes regulated by Hlp and Flp. Intriguingly, our data revealed that Hlp and Flp oppositely regulate the expression of the T3SS, and mutation of *hlp* in the *flp* mutant background restored expression of *hrp* genes and HR induction, but not virulence on host plants, to wild‐type levels (Figure S8). To our knowledge, this is the first report of a pair of NAPs that have opposite effects on the expression of T3SS genes in plant‐pathogenic bacteria but similar effects on virulence. The two NAPs are not functionally interchangeable in terms of regulation of virulence; overexpression of *flp* in the Δ*hlp* mutant does not alter the reduced virulence (Figure S7). The factors that are regulated by Hlp and Flp that contribute to the early phase of invasion remain unknown.

In this work, we focused on the effects of *hlp* mutation on the virulence and T3SS of Xcc. We demonstrated that it plays key roles in these processes. Furthermore, we focused on the interplay between Hlp and Flp and their regulatory effects on the T3SS. However, more work is needed to understand more precisely how Hlp specifically regulates virulence and how the specific interplay between Hlp and Flp affects the expression of T3SS genes in plant‐pathogenic bacteria.

## 4 EXPERIMENTAL PROCEDURES

### 4.1 Bacterial strains, plasmids, and growth conditions

The bacterial strains and plasmids used in this work are listed in Table S1. *E*. *coli* strains were cultured in Luria Bertani medium (Miller, 1972) at 37 °C. Xcc strains were cultured in NYG medium (Daniels et al., 1984), NY medium (NYG medium without glycerol), and the mimic medium XVM2 (Wengelnik & Bonas, 1996) at 28 °C and 200 rpm. Antibiotics were used at the following concentrations as required: kanamycin at 25 μg/ml, rifampicin at 50 μg/ml, ampicillin at 100 μg/ml, spectinomycin at 50 μg/ml, and tetracycline at 5 μg/ml for Xcc strains or 15 μg/ml for *E. coli* strains.

### 4.2 Nucleic acid manipulations

The nucleic acid manipulations followed the procedures described by Sambrook et al. (1989). Conjugation between the Xcc and *E. coli* strains was performed as described by Turner et al. (1985). The restriction endonucleases, T4 DNA ligase, and *Pfu* DNA polymerase were provided by Promega. Total RNA was extracted from cultures of Xcc strains with a total‐RNA extraction kit (Invitrogen) and cDNA was generated using a cDNA synthesis kit (Invitrogen). These kits were used with reference to the manufacturer's instructions. Semiquantitative RT‐PCR and RT‐qPCR were carried out as previously described (Cui et al., 2018; Lu et al., 2009). For semiquantitative RT‐PCR, the obtained cDNA was diluted and used as a template with selected primers for target genes (Table S5). For RT‐qPCR, the SYBR Green‐labelled PCR fragments were amplified using primer sets (Table S5) that were designed based on the transcribed regions of the tested genes. The relative mRNA levels were calculated with respect to the level of the corresponding transcript in the wild‐type strain 8004. The expression level of the 16S rRNA gene was used as an internal standard. The RT‐qPCR tests were performed in triplicate.

### 4.3 Deletion mutant construction and cross‐complementation

The construction of an in‐frame deletion mutant of *hlp* (*XC_1355*) was carried out using a previously described method (Leng et al., 2019). The 439‐bp (*Bam*HI and *Xba*I) upstream sequence and the 340‐bp (*Xba*I and *Hin*dIII) downstream sequence of the *hlp* gene were PCR‐amplified and cloned together into the suicide plasmid pK18*mobsacB* (Schäfer et al., 1994) and transformed into *E. coli* DH5α. The resulting recombined plasmid was introduced into Xcc (wild‐type strain 8004 or *flp* deletion mutant strain Δ*flp*) by triparental mating (using the plasmid pRK2073 as a helper). Mutants were selected and confirmed by PCR and named Δ*hlp* (or Δ*flp*Δ*hlp*). To complement the *hlp* deletion mutant, the recombinant plasmid named pLC*hlp* was introduced into the mutant by triparental mating, resulting in strain CΔ*hlp* (Table 1).

For cross‐complementation of the Δ*flp* mutant, DNA fragments of the *XC_1406*, *XC_1234*, *XC_1355* (*hlp*), *XC_1656*, *XC_1806*, *XC_1860*, *XC_1925*, *XC_3262*, *XC_3597*, *XC_3985*, and *XC_4203* coding sequences were PCR‐amplified from Xcc strain 8004 with the corresponding primer sets (Table S5) and cloned into the vector pLAFR3 to generate the recombinant plasmids pLC1406, pLC1234, pLC*hlp*, pLC1656, pLC1806, pLC1860, pLC1925, pLC3262, pLC3597, pLC3985, and pLC4203, respectively (Table S1). The recombinant plasmids were transferred into the Δ*flp* mutant by triparental conjugation, resulting in strains Δ*flp*/pLC1406, Δ*flp*/pLC1234, Δ*flp*/pLC*hlp*, Δ*flp*/pLC1656, Δ*flp*/pLC1806, Δ*flp*/pLC1860, Δ*flp*/pLC1925, Δ*flp*/pLC3262, Δ*flp*/pLC3597, Δ*flp*/pLC3985, and Δ*flp*/pLC4203 (Table S1).

### 4.4 Pathogenicity tests, in planta growth curve, HR assays, and ion leakage assays

The virulence of Xcc to Chinese radish (*Raphanus sativus*) was tested by the leaf‐clipping method (Dow et al., 2003). Xcc strains, collected from overnight culture, were washed and adjusted to the same final density (OD_600_ = 0.6, approximately 10^9^ cfu/ml). The bacterial resuspension was then diluted to 10^7^ cfu/ml. Leaves were cut with scissors dipped in the bacterial suspensions. The lesions and symptoms were measured 10 days postinoculation.

The growth of bacteria in radish leaf tissue was measured by homogenizing a group of leaves (five leaves for each sample) in 9 ml sterile water. Diluted homogenates were plated on NYG agar plates supplemented with corresponding antibiotics, and bacterial colonies were counted after incubation for 3 days.

HR was tested on pepper leaves (*Capsicum annuum* ‘ECW‐10R’) as previously described (Castañeda et al., 2005; Li et al., 2014). Briefly, bacterial suspensions (10^7^ cfu/ml) were infiltrated into the abaxial side of the pepper leaves. These inoculated plants were kept in the greenhouse to observe the HR symptoms and gauge conductivity at 8, 16, and 24 hr after inoculation. For conductivity measurements, samples (leaf discs of 0.4 cm^2^) were collected and soaked in 10 ml ultrapure water with shaking at 200 rpm. The leaf discs were then removed and the conductivity of water was measured.

### 4.5 Stress tolerance assay

The minimal inhibitory concentration (MIC) method (Li et al., 2020) was employed to test the sensitivity of the Xcc strains to several environmental stresses. Xcc strains were cultured overnight and diluted to an OD_600_ of 0.1. Then 100 μl of the diluted culture was plated on NYG plates supplemented with different concentrations of each reagent. The surviving colonies on the plates were counted after 3 days of incubation at 28 °C.

### 4.6 Exopolysaccharide and extracellular enzyme assays

EPS and extracellular enzyme assays were performed as previously described (Li et al., 2020; Tang et al., 1991). To estimate EPS production, Xcc strains were inoculated into 100 ml NY liquid medium containing glucose (2% wt/vol) at 28 °C, 200 rpm for 3 days. EPS was precipitated from the culture supernatant, dried, and weighed. For estimation of the activity of the extracellular enzymes endoglucanase (cellulase) and amylase, a radial diffusion assay was used as described by Tang et al. (1991). For quantification of endoglucanase and amylase, Xcc strains were cultured in NYG medium for 12 hr. Ten microlitres of enzyme‐containing extracts (supernatant) was added to 200 μl of indicator buffer (citric acid‐Na_2_HPO_4_, pH 5.5) containing 1% (wt/vol) carboxymethylcellulose (for endoglucanase) or 1% (wt/vol) starch solution (for amylase) as the substrate. The reactions were carried out for 30 min at 28 °C. The released reducing sugars were measured as d‐glucose equivalents as described by Miller (1959). One unit (U) of endoglucanase or amylase activity was defined as the amount of enzyme releasing 1 μmol of reducing sugar per minute.

### 4.7 Cell motility assays

Cell motility was detected as previously described (Li et al., 2020). To test swimming motility, two microlitres of bacterial suspension (10^9^ cfu/ml) was stabbed into 0.28% agar plates composed of 0.03% Bacto peptone and 0.03% yeast extract followed by incubation at 28 °C for 4 days. To detect swarming motility, bacterial suspensions were spotted on NY plates containing 2% glucose and 0.6% agar, which were then incubated at 28 °C for 3 days. The diameter of the area occupied by strains was measured and the values were used to indicate the motility of Xcc strains. The experiment was repeated three times.

### 4.8 Transcriptome analysis of the Hlp mutant

Transcriptome analysis was performed as previously described (Cui et al., 2018). Briefly, Xcc strains were cultured in XVM2 medium to a concentration of OD_600_ of 0.6. RNA was prepared and the contaminating genomic DNA was removed. After the quantity was determined and quality was assessed, total RNA was sent to Novogene (Beijing, China) for library construction and strand‐specific RNA sequencing. Clean reads were mapped to the genome of Xcc strain 8004 and the reads per kilobase per million mapped reads method was used to calculate the gene expression levels. Genes with false discovery rate ≤ .05 and |log_2_(fold change)| ≥ 1 were considered for differentially expressed.

## Supporting information


**FIGURE S1** Disease symptoms caused by *Xanthomonas campestris* pv. *campestris* (Xcc) wild‐type strain 8004, the *hlp* deletion mutant Δ*hlp*, the complemented strain CΔ*hlp*, and control strain Δ*hlp*/pLAFR3. Sterile water was used for mock inoculation. Xcc strains were inoculated onto the leaves of Chinese radish by the leaf‐clipping method. Ten days after inoculation, representative infected leaves for each Xcc strain were photographed

**FIGURE S2** Growth of *Xanthomonas campestris* pv. *campestris* (Xcc) strains in mimic medium. The strains were inoculated into 100 ml XVM2 medium, which mimics more closely the nutrition environment of the plant. Samples were taken in triplicate at intervals of 4 hr, diluted, and plated on NYG plates

**FIGURE S3** Sequence alignments between Hlp and HU_Bst_ proteins in *Escherichia coli* (accession number WP_160456856) and *Geobacillus stearothermophilus* (accession number AAA22532). The sequences of these proteins were acquired from NCBI and alignment was performed with the software NTI Vector. Residues that are identical in three and two sequences are shown with a red and blue background, respectively

**FIGURE S4** Stress tolerances in tested *Xanthomonas campestris* pv. *campestris* (Xcc) strains. Survival experiments were performed by subculturing strains overnight on fresh NYG agar plates supplemented with different concentrations of NaCl (i), H_2_O_2_ (ii), phenol (iii), CuSO_4_ (iv), CdCl_2_ (v), and ZnSO_4_ (vi). The surviving bacterial colonies on the plates were counted after incubation for 3 days

**FIGURE S5** The levels of extracellular enzymes produced by the Δ*hlp* mutant were similar to those of the wild type. (a) Radial diffusion assays for the activity of extracellular enzymes of *Xanthomonas campestris* pv. *campestris* (Xcc) strains. An overnight culture (2 μl, OD_600_ = 1.0) of each Xcc strain was spotted onto NYG plates containing 0.25% (wt/vol) carboxymethylcellulose (for endoglucanase) or 0.1% (wt/vol) starch (for amylase) and incubated at 28 °C for 24 hr. Plates were stained as described by Tang et al. (1991). Zones of clearance around the spot due to degradation of the substrate were photographed. At least three plates were inoculated in each experiment and each experiment was repeated three times. (b) Quantification of extracellular enzymes produced by Xcc strains. Xcc strains were cultured in NYG medium for 12 hr, and the activities of endoglucanase (cellulase) and amylase were measured as previously described (Li et al., 2020). Ten microlitres of enzyme‐containing extracts was added to 200 μl of indicator buffer containing 1% (wt/vol) carboxymethylcellulose (for endoglucanase) or 1% (wt/vol) starch solution (for amylase) as the substrate. The reactions were carried out for 30 min at 28 °C. The released reducing sugars were measured as d‐glucose equivalents. One unit (U) of endoglucanase/amylase activity was defined as the amount of enzyme releasing 1 μmol of reducing sugar per minute. Data are shown as the mean ± *SD* of triplicate measurements from a representative experiment; analysis of variance and Dunnett's post hoc test were used to identify significant differences. No groups were statistically different from the wild type (*p* > .05 for all comparisons). n.s., not significant. The experiment was repeated twice and similar results were obtained

**FIGURE S6** Disease symptoms caused by *Xanthomonas campestris* pv. *campestris* (Xcc) wild‐type strain 8004, the *flp* deletion mutant Δ*flp*, cross‐complemented strains (Δ*flp*/pLC*hlp*, Δ*flp*/pLC1046, and Δ*flp*/pLC1234), and the complemented strain CΔ*flp*. Xcc strains were inoculated onto the leaves of Chinese radish by the leaf‐clipping method. Ten days after inoculation, representative infected leaves for each Xcc strain were photographed

**FIGURE S7** Overexpression of the *flp* gene in the Δ*hlp* mutant cannot restore its phenotypes. The recombinant plasmid pLC*flp*, which was generated by cloning the *flp* gene cloned into the vector pLAFR3 (Leng et al., 2019), was transferred into the Δ*hlp* mutant, obtaining strain Δ*hlp*/pLC*flp*. *Xanthomonas campestris* pv. *campestris* (Xcc) wild‐type strain 8004, the Δ*hlp* mutant, and the cross‐complemented strain Δ*hlp*/pLC*flp* were cultured in NYG medium overnight and adjusted to appropriate concentrations in sterile distilled water or NYG medium. (a) Lengths of lesions caused by Xcc strains. Xcc strains were inoculated into Chinese radish by the leaf‐clipping method. Lesion lengths were scored 10 days post‐inoculation. Data are shown as the mean ± *SD* from 15 inoculated leaves in one experiment. Analysis of variance (ANOVA) and Dunnett's post hoc test were used to identify significant differences (**p < .01; n.s., not significant). The experiment was repeated twice, and similar results were obtained. (b) Extracellular polysaccharide (EPS) yield of tested Xcc strains. Xcc strains were subcultured in NY medium containing 2% glucose for 3 days before EPS was extracted and quantified. Data are shown as the mean ± *SD* of three replicates from a representative experiment. ANOVA and Dunnett's post hoc test were used to identify significant differences (*p < .05; n.s., not significant). The experiment was repeated twice with similar results. (c) Cell motilities of tested Xcc strains. Two microlitres of culture suspension (10^9^ cfu/ml) of Xcc strains was stabbed into “swim” (0.28% agar) medium and incubated for 4 days at 28 °C or inoculated onto “swarm” (0.6% agar) plates and incubated for 3 days at 28 °C. The colony morphologies were photographed

**FIGURE S8** Virulence test of different *Xanthomonas campestris* pv. *campestris* (Xcc) strains. The Xcc wild‐type strain 8004, the *hlp*/*flp* double deletion mutant strain Δ*flp*Δ*hlp*, the *flp* mutant strain Δ*flp*, and the *hlp* mutant strain Δ*hlp* were inoculated onto the leaves of Chinese radish by the leaf‐clipping method. Ten days after inoculation, (a) disease symptoms for each Xcc strain were photographed and (b) lesion lengths were scored. Values given are the mean and *SD* from 15 inoculated leaves in one experiment. Significance was determined by analysis of variance and Dunnett's post hoc test for comparison with the wild type. **p < .01; n.s., not significant. The experiment was repeated three times with similar results

**TABLE S1** Bacterial strains and plasmids used in this work

**TABLE S2** Genes expressed by the Δ*hlp* mutant strain when grown in XVM2

**TABLE S3** Confirmation of RNA‐seq gene expression data by semiquantitative reverse‐transcription PCR

**TABLE S4** Genes coregulated by both Hlp and Flp

**TABLE S5** Primers used in this study

## Data Availability

The data that support the findings of this study are available from the corresponding author upon reasonable request.
