# Supplementary figures and images for: A HU‐like protein is required for full virulence in Xanthomonas campestris pv. campestris
Source: Mol Plant Pathol. 2021 Aug 23;22(12):1574–86. doi: 10.1111/mpp.13128 (PMC8578834; doi:10.1111/mpp.13128)

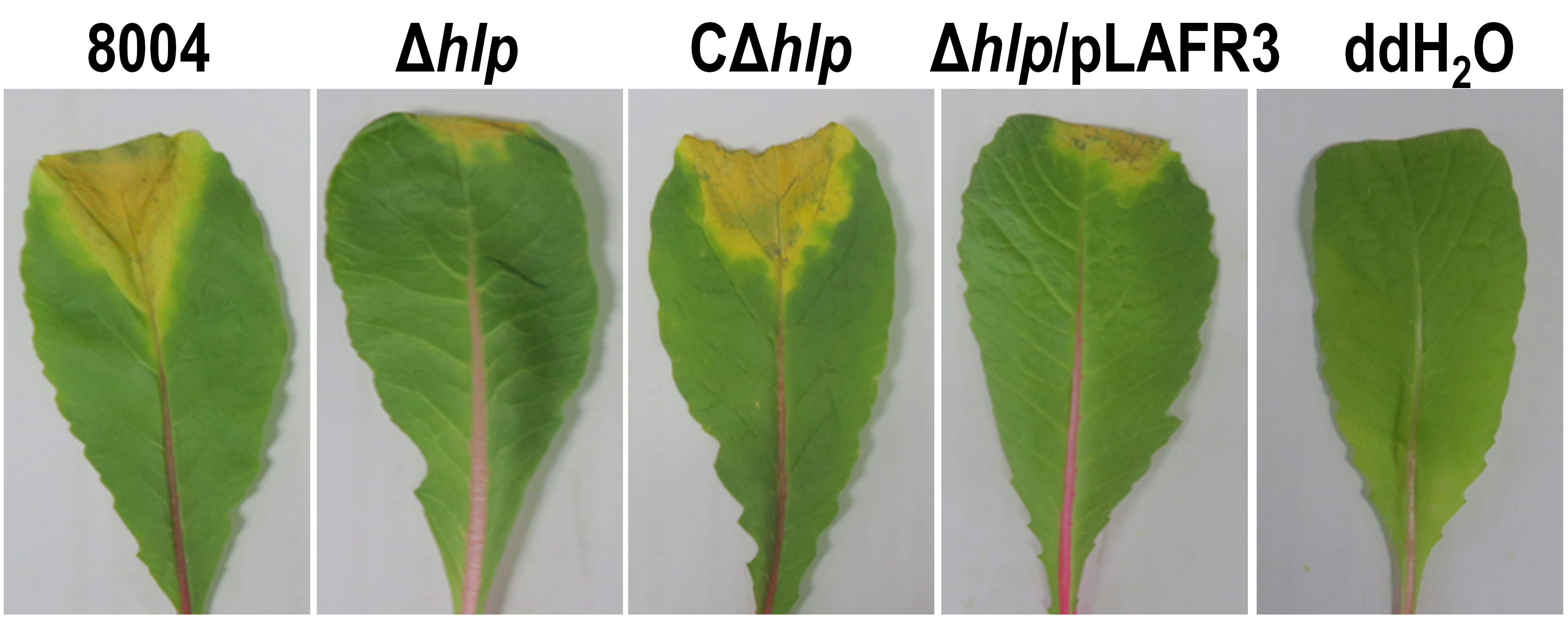

Supplement: Supplementary file 1 — FIGURE S1 Disease symptoms caused by Xanthomonas campestris pv. campestris (Xcc) wild‐type strain 8004, the hlp deletion mutant Δhlp, the complemented strain CΔhlp, and control strain Δhlp/pLAFR3. Sterile water was used for mock inoculation. Xcc strains were inoculated onto the leaves of Chinese radish by the leaf‐clipping method. Ten days after inoculation, representative infected leaves for each Xcc strain were photographed [file MPP-22-1574-s010.jpg]

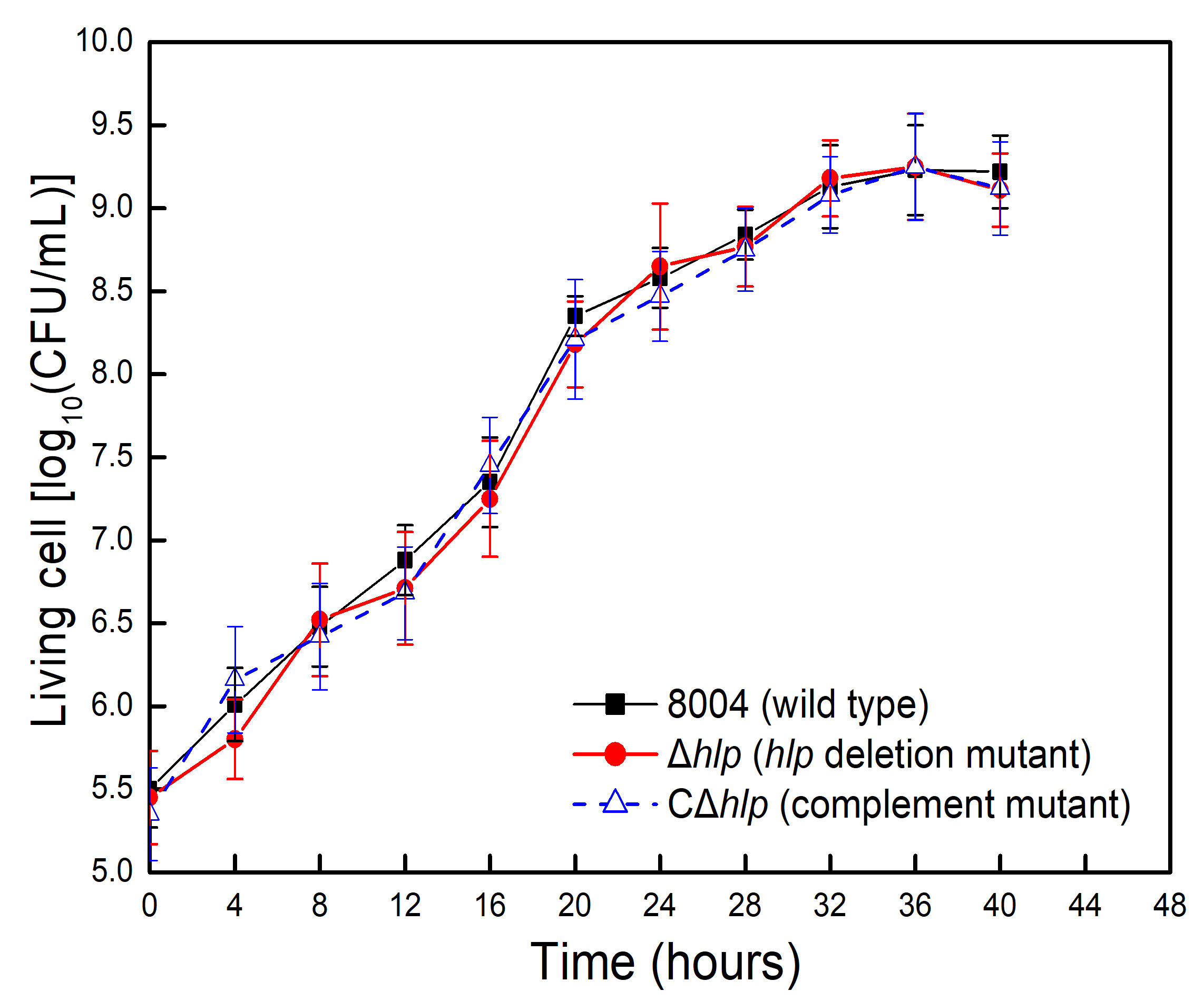

Supplement: Supplementary file 2 — FIGURE S2 Growth of Xanthomonas campestris pv. campestris (Xcc) strains in mimic medium. The strains were inoculated into 100 ml XVM2 medium, which mimics more closely the nutrition environment of the plant. Samples were taken in triplicate at intervals of 4 hr, diluted, and plated on NYG plates [file MPP-22-1574-s007.jpg]

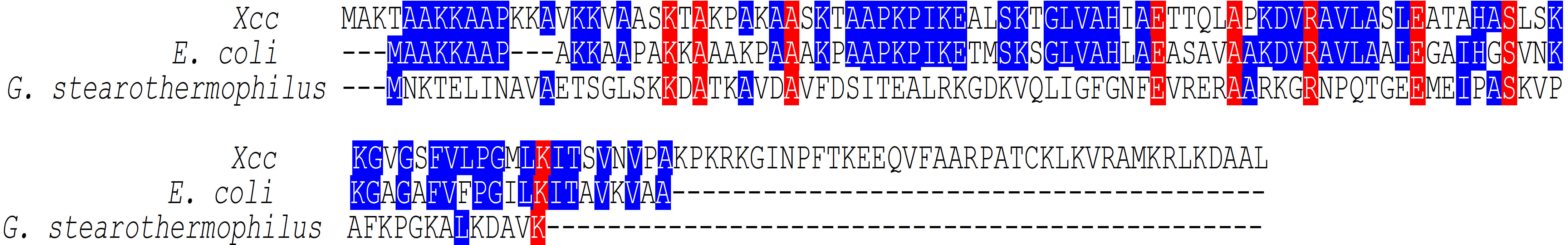

Supplement: Supplementary file 3 — FIGURE S3 Sequence alignments between Hlp and HUBst proteins in Escherichia coli (accession number WP_160456856) and Geobacillus stearothermophilus (accession number AAA22532). The sequences of these proteins were acquired from NCBI and alignment was performed with the software NTI Vector. Residues that are identical in three and two sequences are shown with a red and blue background, respectively [file MPP-22-1574-s009.jpg]

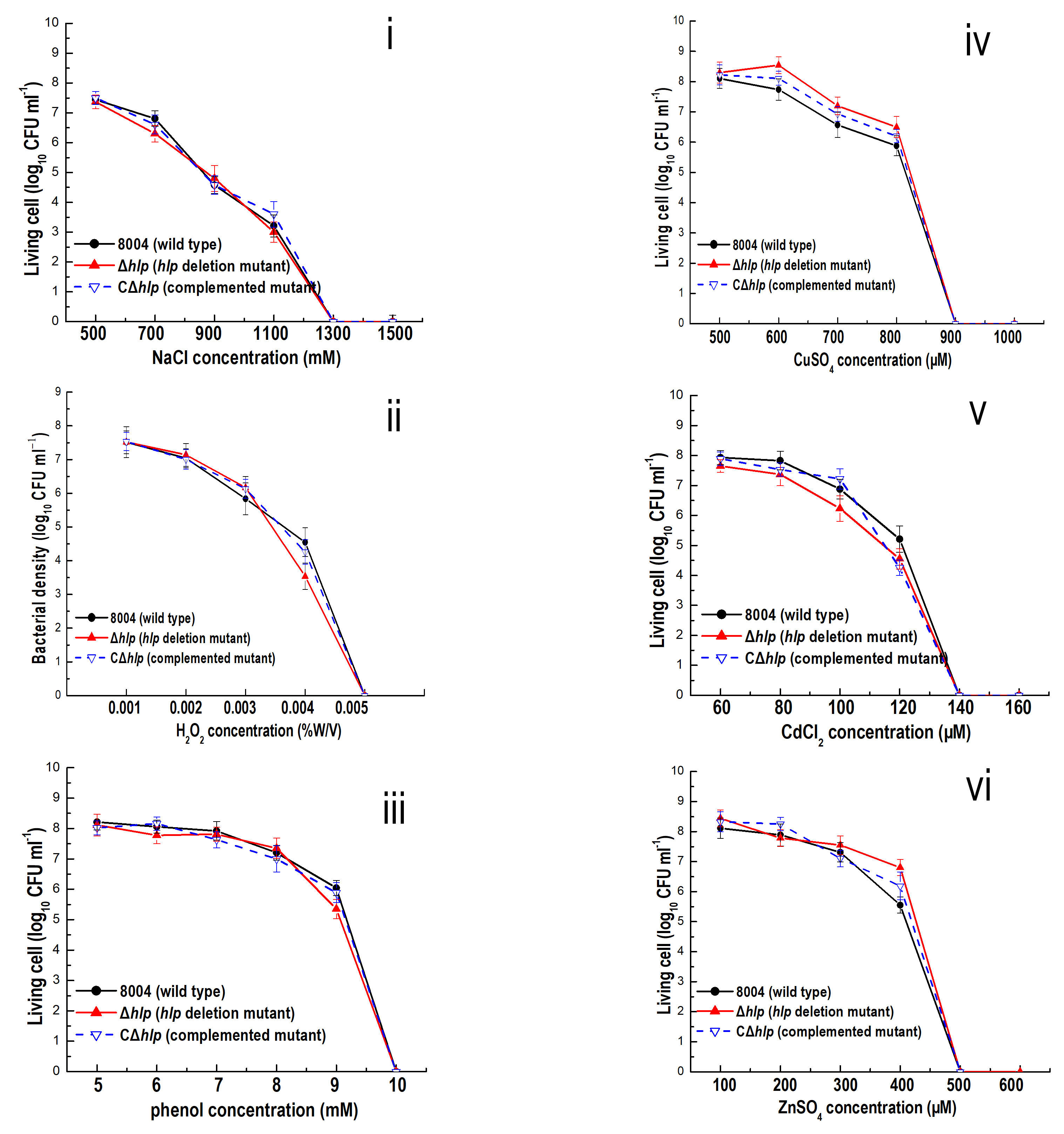

Supplement: Supplementary file 4 — FIGURE S4 Stress tolerances in tested Xanthomonas campestris pv. campestris (Xcc) strains. Survival experiments were performed by subculturing strains overnight on fresh NYG agar plates supplemented with different concentrations of NaCl (i), H2O2 (ii), phenol (iii), CuSO4 (iv), CdCl2 (v), and ZnSO4 (vi). The surviving bacterial colonies on the plates were counted after incubation for 3 days [file MPP-22-1574-s001.jpg]

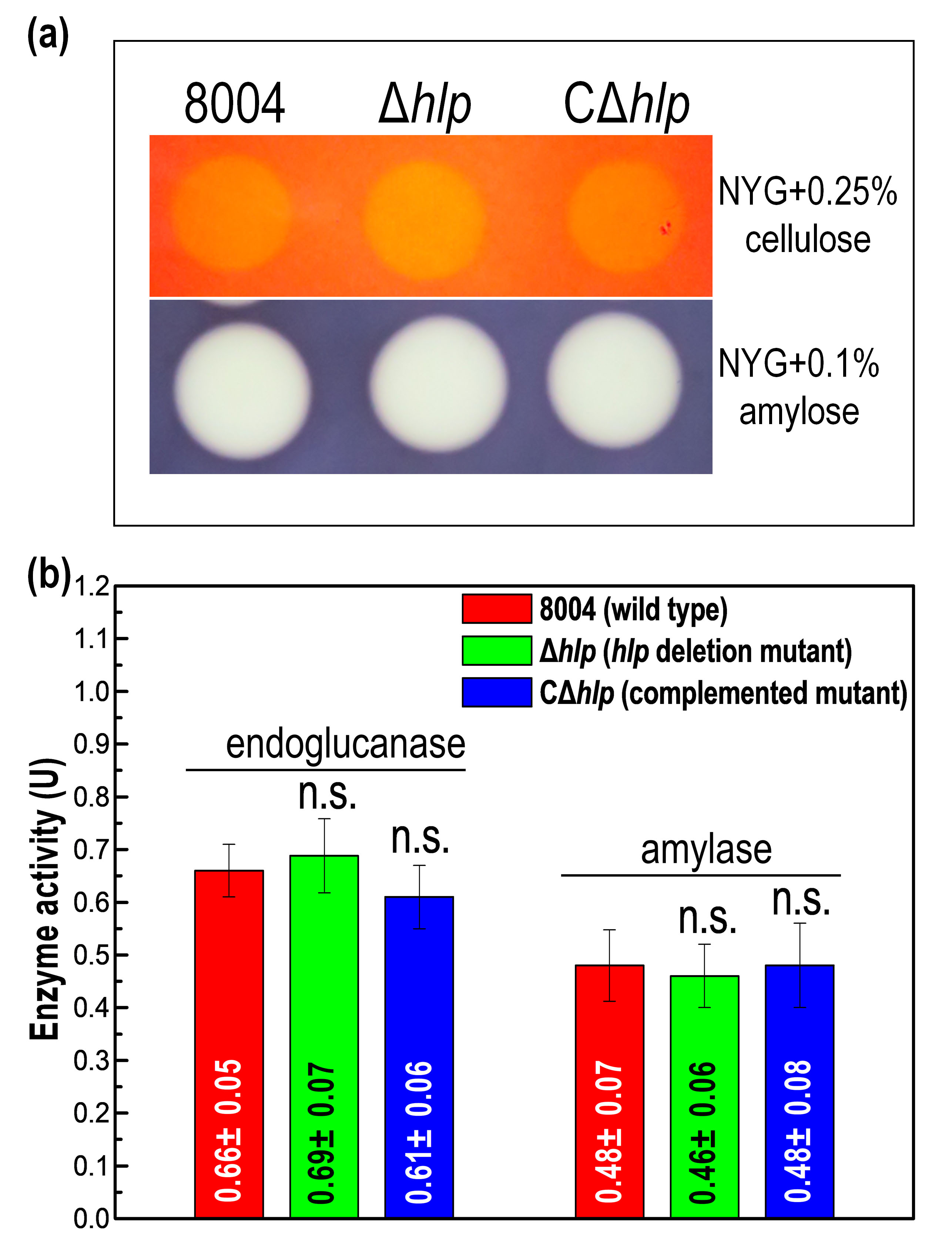

Supplement: Supplementary file 5 — FIGURE S5 The levels of extracellular enzymes produced by the Δhlp mutant were similar to those of the wild type. (a) Radial diffusion assays for the activity of extracellular enzymes of Xanthomonas campestris pv. campestris (Xcc) strains. An overnight culture (2 μl, OD600 = 1.0) of each Xcc strain was spotted onto NYG plates containing 0.25% (wt/vol) carboxymethylcellulose (for endoglucanase) or 0.1% (wt/vol) starch (for amylase) and incubated at 28 °C for 24 hr. Plates were stained as described by Tang et al. (1991). Zones of clearance around the spot due to degradation of the substrate were photographed. At least three plates were inoculated in each experiment and each experiment was repeated three times. (b) Quantification of extracellular enzymes produced by Xcc strains. Xcc strains were cultured in NYG medium for 12 hr, and the activities of endoglucanase (cellulase) and amylase were measured as previously described (Li et al., 2020). Ten microlitres of enzyme‐containing extracts was added to 200 μl of indicator buffer containing 1% (wt/vol) carboxymethylcellulose (for endoglucanase) or 1% (wt/vol) starch solution (for amylase) as the substrate. The reactions were carried out for 30 min at 28 °C. The released reducing sugars were measured as d‐glucose equivalents. One unit (U) of endoglucanase/amylase activity was defined as the amount of enzyme releasing 1 μmol of reducing sugar per minute. Data are shown as the mean ± SD of triplicate measurements from a representative experiment; analysis of variance and Dunnett's post hoc test were used to identify significant differences. No groups were statistically different from the wild type (p > .05 for all comparisons). n.s., not significant. The experiment was repeated twice and similar results were obtained [file MPP-22-1574-s011.jpg]

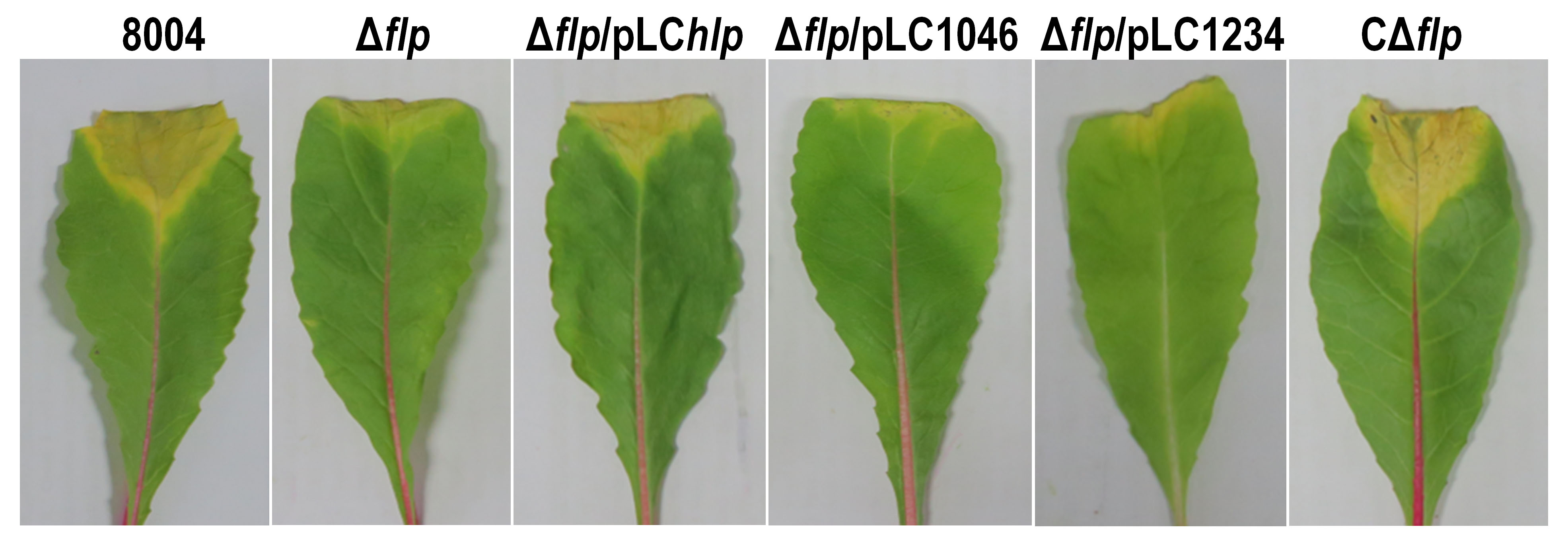

Supplement: Supplementary file 6 — FIGURE S6 Disease symptoms caused by Xanthomonas campestris pv. campestris (Xcc) wild‐type strain 8004, the flp deletion mutant Δflp, cross‐complemented strains (Δflp/pLChlp, Δflp/pLC1046, and Δflp/pLC1234), and the complemented strain CΔflp. Xcc strains were inoculated onto the leaves of Chinese radish by the leaf‐clipping method. Ten days after inoculation, representative infected leaves for each Xcc strain were photographed [file MPP-22-1574-s005.jpg]

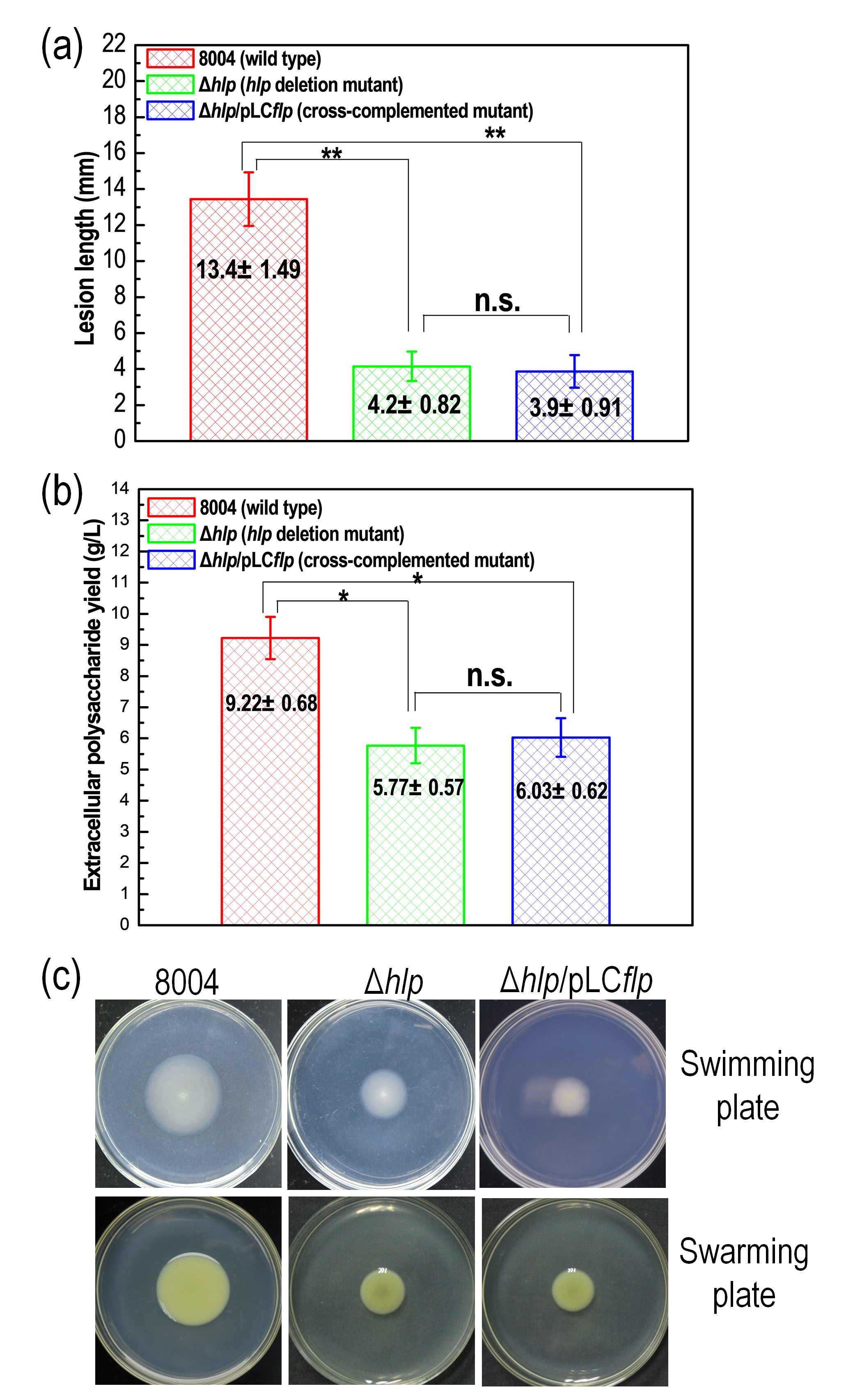

Supplement: Supplementary file 7 — FIGURE S7 Overexpression of the flp gene in the Δhlp mutant cannot restore its phenotypes. The recombinant plasmid pLCflp, which was generated by cloning the flp gene cloned into the vector pLAFR3 (Leng et al., 2019), was transferred into the Δhlp mutant, obtaining strain Δhlp/pLCflp. Xanthomonas campestris pv. campestris (Xcc) wild‐type strain 8004, the Δhlp mutant, and the cross‐complemented strain Δhlp/pLCflp were cultured in NYG medium overnight and adjusted to appropriate concentrations in sterile distilled water or NYG medium. (a) Lengths of lesions caused by Xcc strains. Xcc strains were inoculated into Chinese radish by the leaf‐clipping method. Lesion lengths were scored 10 days post‐inoculation. Data are shown as the mean ± SD from 15 inoculated leaves in one experiment. Analysis of variance (ANOVA) and Dunnett's post hoc test were used to identify significant differences (**p < .01; n.s., not significant). The experiment was repeated twice, and similar results were obtained. (b) Extracellular polysaccharide (EPS) yield of tested Xcc strains. Xcc strains were subcultured in NY medium containing 2% glucose for 3 days before EPS was extracted and quantified. Data are shown as the mean ± SD of three replicates from a representative experiment. ANOVA and Dunnett's post hoc test were used to identify significant differences (*p < .05; n.s., not significant). The experiment was repeated twice with similar results. (c) Cell motilities of tested Xcc strains. Two microlitres of culture suspension (109 cfu/ml) of Xcc strains was stabbed into “swim” (0.28% agar) medium and incubated for 4 days at 28 °C or inoculated onto “swarm” (0.6% agar) plates and incubated for 3 days at 28 °C. The colony morphologies were photographed [file MPP-22-1574-s003.jpg]

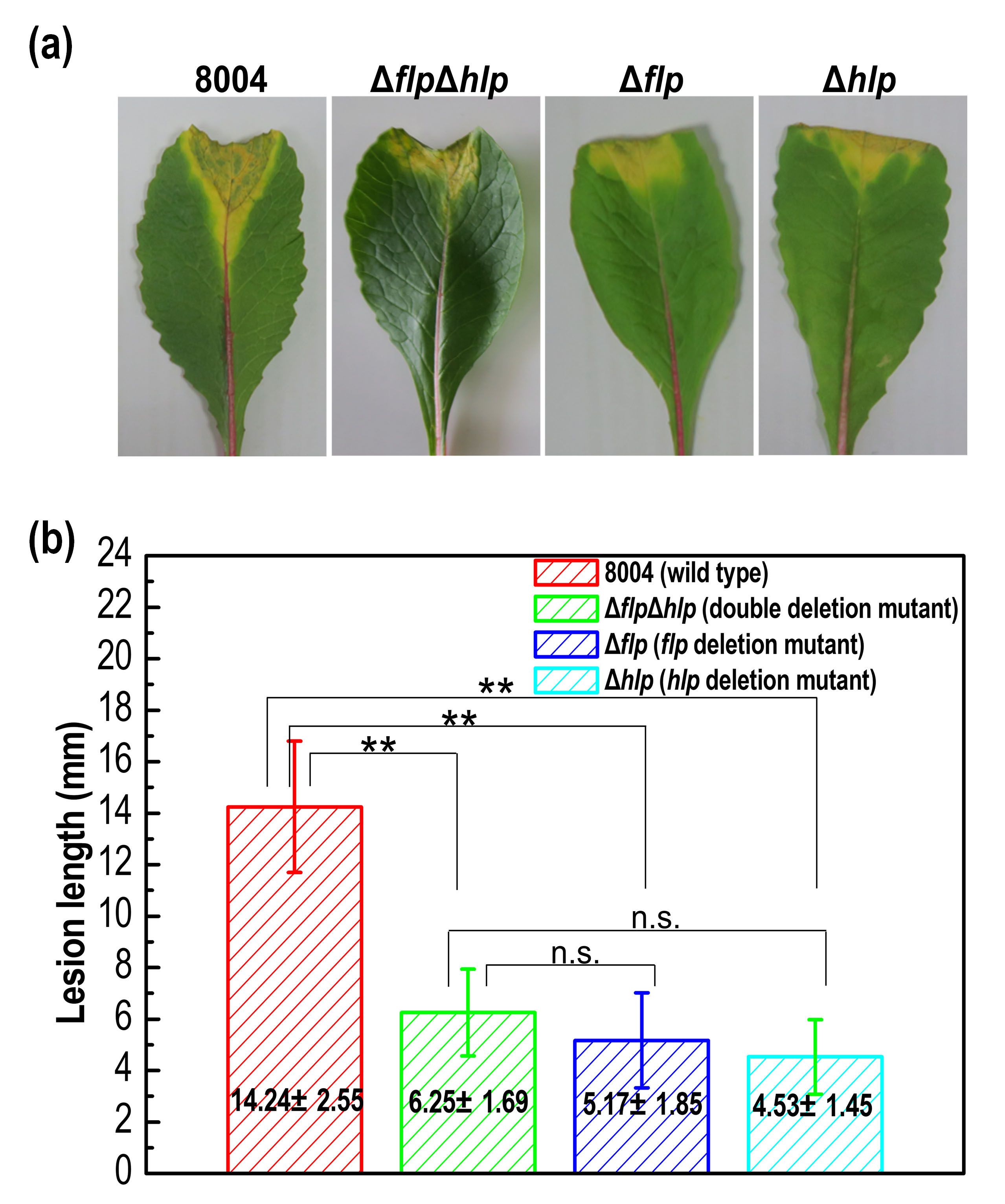

Supplement: Supplementary file 8 — FIGURE S8 Virulence test of different Xanthomonas campestris pv. campestris (Xcc) strains. The Xcc wild‐type strain 8004, the hlp/flp double deletion mutant strain ΔflpΔhlp, the flp mutant strain Δflp, and the hlp mutant strain Δhlp were inoculated onto the leaves of Chinese radish by the leaf‐clipping method. Ten days after inoculation, (a) disease symptoms for each Xcc strain were photographed and (b) lesion lengths were scored. Values given are the mean and SD from 15 inoculated leaves in one experiment. Significance was determined by analysis of variance and Dunnett's post hoc test for comparison with the wild type. **p < .01; n.s., not significant. The experiment was repeated three times with similar results [file MPP-22-1574-s012.jpg]
