# Supplementary material for: A HU‐like protein is required for full virulence in Xanthomonas campestris pv. campestris
Source: Mol Plant Pathol. 2021 Aug 23;22(12):1574–86. doi: 10.1111/mpp.13128 (PMC8578834; doi:10.1111/mpp.13128)
Supplement: Supplementary file 9 — TABLE S1 Bacterial strains and plasmids used in this work [file MPP-22-1574-s006.docx]

**Table S1**. Bacterial strains and plasmids used in this work

| Strains or plasmids | Relevant characteristics | Reference or source |
| --- | --- | --- |
| *E. coli* strains |  |  |
| DH5α | Φ80△*lacZM*15 *recA1 endA1 deoR* | Gibco BRL, Life Technologies |
| *Xanthomonas campestris* pv. *campestris* strains |  |  |
| 8004 | Wild-type strain, Rif^r^ | Daniels *et al.*, 1984 |
| 1046pk | As 8004, but *XC_1046*::pK18*mob*, polar effect. Rif^r^ Kan^r^. | Author’s lab collection |
| 195B09 | As 8004, but *XC_1234*::Tn5*gusA*5, polar effect. Rif^r^ Kan^r^. | Author’s lab collection |
| 1355pk | As 8004, but *XC_1355*::pK18*mob*, polar effect. Rif^r^ Kan^r^. | Author’s lab collection |
| 1656pk | As 8004, but *XC_1656*::pK18*mob*, polar effect. Rif^r^ Kan^r^. | Author’s lab collection |
| 1806pk | As 8004, but *XC_1806*::pK18*mob*, polar effect. Rif^r^ Kan^r^. | Author’s lab collection |
| 1860nk | As 8004, but *XC_1860*::pK18*mob*, non-polar effect. Rif^r^ Kan^r^. | Author’s lab collection |
| 247H03 | As 8004, but *XC_1925*::Tn5*gusA*5, polar effect. Rif^r^ Kan^r^. | Author’s lab collection |
| 3262nk | As 8004, but *XC_3262*::pK18*mob*, non-polar effect. Rif^r^ Kan^r^. | Author’s lab collection |
| 100B06 | As 8004, but *XC_3597*::Tn5*gusA*5, polar effect. Rif^r^ Kan^r^. | Author’s lab collection |
| 147G06 | As 8004, but *XC_3985*::Tn5*gusA*5, polar effect. Rif^r^ Kan^r^. | Author’s lab collection |
| 4203pk | As 8004, but *XC_4203*::pK18*mob*, polar effect. Rif^r^ Kan^r^. | Author’s lab collection |
| Δ*flp* | As 8004, but *flp* gene (*XC_0520*) deleted, non-polar effect. Rif^r^ | Leng *et al*., 2019 |
| C∆*flp* | ∆*flp* harboring the recombinant plasmid pLC*flp*. Rif^r^ Tet^r^ | Leng *et al*., 2019 |
| Δ*flp*/pLC*hlp* | ∆*flp* harboring the recombinant plasmid pLC*hlp*. Rif^r^ Tet^r^ | This work |
| Δ*flp*/pLC1046 | ∆*flp* harboring the recombinant plasmid pLC1046. Rif^r^ Tet^r^ | This work |
| Δ*flp*/pLC1234 | ∆*flp* harboring the recombinant plasmid pLC1234. Rif^r^ Tet^r^ | This work |
| Δ*flp*/pLC1656 | ∆*flp* harboring the recombinant plasmid pLC1656. Rif^r^ Tet^r^ | This work |
| Δ*flp*/pLC1806 | ∆*flp* harboring the recombinant plasmid pLC1806. Rif^r^ Tet^r^ | This work |
| Δ*flp*/pLC1860 | ∆*flp* harboring the recombinant plasmid pLC1860. Rif^r^ Tet^r^ | This work |
| Δ*flp*/pLC1925 | ∆*flp* harboring the recombinant plasmid pLC1925. Rif^r^ Tet^r^ | This work |
| Δ*flp*/pLC3262 | ∆*flp* harboring the recombinant plasmid pLC3262. Rif^r^ Tet^r^ | This work |
| Δ*flp*/pLC3597 | ∆*flp* harboring the recombinant plasmid pLC3597. Rif^r^ Tet^r^ | This work |
| Δ*flp*/pLC3985 | ∆*flp* harboring the recombinant plasmid pLC3985. Rif^r^ Tet^r^ | This work |
| Δ*flp*/pLC4203 | ∆*flp* harboring the recombinant plasmid pLC4203. Rif^r^ Tet^r^ | This work |
| Δ*hlp* | As 8004, but *hlp* gene (*XC_1355*) deleted, non-polar effect. Rif^r^ | This work |
| C∆*hlp* | ∆*hlp* harboring the recombinant plasmid pLC*hlp*. Rif^r^ Tet^r^ | This work |
| Δ*hlp*/pLC*flp* | ∆*hlp* harboring the recombinant plasmid pLC*flp*. Rif^r^ Tet^r^ | This work |
| Δ*flp*Δ*hlp* | As 8004, but both *flp* gene and *hlp* deleted, non-polar effect. Rif^r^ | This work |
| Plasmids |  |  |
| pLAFR3 | Broad host range cloning vector. Tet^r^ | Staskawicz *et al*.,1987 |
| pLC1046 | pLAFR3 containing an 390-bp DNA fragment of the ORF *XC_1046* of *Xcc* strain. Tet^r^ | This work |
| pLC1234 | pLAFR3 containing an 468-bp DNA fragment of the ORF *XC_1234* of *Xcc* strain. Tet^r^ | This work |
| pLC*hlp* | pLAFR3 containing an 420-bp DNA fragment of the *hlp* gene (*XC_1355*) of *Xcc* strain. Tet^r^ | This work |
| pLC1656 | pLAFR3 containing an 297-bp DNA fragment of the ORF *XC_1656* of *Xcc* strain. Tet^r^ | This work |
| pLC1806 | pLAFR3 containing an 402-bp DNA fragment of the ORF *XC_1806* of *Xcc* strain. Tet^r^ | This work |
| pLC1860 | pLAFR3 containing an 258-bp DNA fragment of the ORF *XC_1860* of *Xcc* strain. Tet^r^ | This work |
| pLC1925 | pLAFR3 containing an 309-bp DNA fragment of the ORF *XC_1925* of *Xcc* strain. Tet^r^ | This work |
| pLC3262 | pLAFR3 containing an 270-bp DNA fragment of the ORF *XC_3262* of *Xcc* strain. Tet^r^ | This work |
| pLC3597 | pLAFR3 containing an 465-bp DNA fragment of the ORF *XC_3597* of *Xcc* strain. Tet^r^ | This work |
| pLC3985 | pLAFR3 containing an 777-bp DNA fragment of the ORF *XC_3985* of *Xcc* strain. Tet^r^ | This work |
| pLC4203 | pLAFR3 containing an 249-bp DNA fragment of the ORF *XC_4203* of *Xcc* strain. Tet^r^ | This work |
| pLC*flp* | pLAFR3 containing an 297-bp DNA fragment of the *flp* gene (*XC_0520*) of *Xcc* strain. Tet^r^ | Leng *et al*., 2019 |
| pRK2073 | Helper plasmid, Tra^+^, Mob^+^, ColE1, Spc^r^. | Leong *et al*.,1982 |
| pK18*mob* | pUC18 derivative, *lacZα* Kan^r^, *mob* site. Suicide plasmid in *Xcc*. | Schäfer *et al.*, 1994 |
| pK18*mobsacB* | pUC18 derivative, *lacZα*, *sacB*, Kan^r^, *mob* site. Allelic exchange vector (Suicidal vector carrying *sacB* gene for mutagenesis). | Schäfer *et al*., 1994 |
| pKΔ*hlp* | pK18*mobsacB* containing fragments flanking *hlp* gene. Kan^r^ | This work |

^a^Rif^r^, Kan^r^, Tet^r^, Ampr and Spc^r^ indicate resistance to rifampicin, kanamycin, tetracycline, ampicillin and spectinomycin, respectively.

**References**

Daniels, M.J., Barber, C.E., Turner, P.C., Sawczyc, M.K., Byrde, R.J.W., and Fielding, A.H. (1984) Cloning of genes involved in pathogenicity of *Xanthomonas campestris* pv. *campestris* using the broad host range cosmid pLAFR1. *EMBO J.* 3, 3323–3328.

Leng, M., Lu, Z.J., Qin, Z.S., Qi, Y.H., Lu, G.T. and Tang, J.L. (2019) Flp, a Fis-like protein, contributes to the regulation of type III secretion and virulence processes in the phytopathogen Xanthomonas campestris pv. campestris. *Molecular plant pathology* 20, 1119–1133.

Leong, S.A., Ditta, G.S., and Helinski, D.R. (1982) Heme biosynthesis in *Rhizobium*. Identification of a cloned gene coding for delta-aminolevulinic acid synthetase from *Rhizobium meliloti*. *J. Biol. Chem.* 257, 8724–8730.

Schäfer, A., Tauch, A., Jäger, W., Kalinowski, J., Thierbach, G., and Pühler, A. (1994) Small mobilizable multi-purpose cloning vectors derived from the *Escherichia coli* plasmids pK18 and pK19: selection of defined deletions in the chromosome of *Corynebacterium glutamicum*. *Gene* 145, 69–73.

Staskawicz, B., Dahlbeck, D., Keen, N., and Napoli, C. (1987) Molecular characterization of cloned avirulence genes fromrace 0 and race 1 of *Pseudomonas syringae* pv. *glycinea*. *J. Bacteriol.* 169, 5789–5794.
