# Supplementary material for: A HU‐like protein is required for full virulence in Xanthomonas campestris pv. campestris
Source: Mol Plant Pathol. 2021 Aug 23;22(12):1574–86. doi: 10.1111/mpp.13128 (PMC8578834; doi:10.1111/mpp.13128)
Supplement: Supplementary file 10 — TABLE S2 Genes expressed by the Δhlp mutant strain when grown in XVM2 [file MPP-22-1574-s013.docx]

**Table S2.** Genes expressed by the Δ*hlp* mutant strain when grown in XVM2.

| Function Category | Gene ID | Name | Annotation | fold change |
| --- | --- | --- | --- | --- |
| Amino acid biosynthesis | *XC_0330* | *metE* | 5-methyltetrahydropteroyltriglutamate-homocysteine methyltransferase | 28.27 |
|  | *XC_0473* | *trpI* | anthranilate synthase component I | 3.64 |
|  | *XC_0481* | *trpG* | nthranilate synthase | 4.01 |
|  | *XC_0483* | *trpD* | anthranilate synthase | 4.86 |
|  | *XC_0548* | *aroG* | 3-deoxy-7-phosphoheptulonate synthase | 3.59 |
|  | *XC_0662* | *dapF* | diaminopimelate epimerase | 3.11 |
|  | *XC_0840* | *ilvB* | acetolactate synthase | 3.88 |
|  | *XC_0841* | *ilvC* | ketol-acid reductoisomerase | 4.85 |
|  | *XC_0882* | *argD* | acetylornithine aminotransferase | 2.5 |
|  | *XC_0889* | *hisC* | histidinol-phosphate aminotransferase | 3.05 |
|  | *XC_1090* | *metB* | homocysteine synthase | 6.04 |
|  | *XC_1103* | *lysA* | diaminopimelate decarboxylase | 13.27 |
|  | *XC_1251* | *metX* | homoserine O-acetyltransferase | 4.52 |
|  | *XC_1252* | *metB* | cystathionine gamma-synthase | 5.19 |
|  | *XC_1569* | *Asd* | aspartate semialdehyde dehydrogenase | 2.89 |
|  | *XC_2177* | *nirB* | nitrite reductase (NADH) large subunit | 3.55 |
|  | *XC_2724* | *metH* | 5-methyltetrahydrofolate-homocysteine methyl transferase | 3.33 |
|  | *XC_2725* | *metH* | 5-methyltetrahydrofolate-homocysteine methyltransferase | 2.88 |
|  | *XC_2848* | *asnB* | asparagine synthase B | 7 |
|  | *XC_3030* | *cefD* | isopenicillin-N epimerase | 5.56 |
|  | *XC_2760* | *tyrA* | chorismate mutase/prephenate dehydrogenase | -2.72 |
| Biosynthesis of cofactors, prosthetic groups, carriers | *XC_0012* | *pdxJ* | pyridoxal phosphate biosynthetic protein | 4.86 |
|  | *XC_0301* | *ggt* | gamma-glutamyltranspeptidase | 4.38 |
|  | *XC_0842* | *ggt* | gamma-glutamyltranspeptidase | 2.53 |
|  | *XC_0886* | *aofH* | monoamine oxidase | 5.06 |
|  | *XC_0895* | *thiE* | thiamin-phosphate pyrophosphorylase | 3.01 |
|  | *XC_0983* | *cysG* | siroheme synthase | 4.62 |
|  | *XC_1094* | *cobA* | cob(I)alamin adenosyltransferase | 3.95 |
|  | *XC_3552* | *pncB* | nicotinate phosphoribosyltransferase | 2.42 |
|  | *XC_3952* | *entF* | enterobactin synthetase | 3.02 |
|  | *XC_0576* | *adcG* | phosphoribosyl-dephospho-CoA transferase | -2.83 |
| Cell envelope and cell structure | *XC_0188* | *plsC* | acetyltransferase | 3.33 |
|  | *XC_0228* |  | AMP-ligase | 4.32 |
|  | *XC_0504* |  | transmembrane protein | 4.78 |
|  | *XC_0609* |  | inner membrane protein | 9.05 |
|  | *XC_0685* |  | surface antigen gene | 4.78 |
|  | *XC_0802* |  | integral membrane nucleotide protein | 2.91 |
|  | *XC_0893* | *oar* | Oar protein | 6.06 |
|  | *XC_0894* |  | Oar protein | 4.53 |
|  | *XC_0940* | *pilN* | type IV pilus assembly protein PilN | 3.58 |
|  | *XC_0941* | *pilM* | fimbrial assembly membrane protein | 5.02 |
|  | *XC_0969* |  | outer membrane protein | 18.5 |
|  | *XC_0970* | *ompW* | outer membrane protein | 9.26 |
|  | *XC_1058* | *pilA* | type IV pilus assembly protein PilA | 2.82 |
|  | *XC_1142* | *pal* | peptidoglycan-associated lipoprotein | 3.78 |
|  | *XC_1183* | *pilG* | two-component system response regulator PilG | 3.8 |
|  | *XC_1184* | *pilH* | two-component system response regulator PilH | 6.09 |
|  | *XC_1186* | *pilJ* | pilus biogenesis protein | 5.47 |
|  | *XC_1187* | *pilL* | PilL protein,pilin biosynthetic protein | 4.74 |
|  | *XC_1219* |  | beta-hexosaminidase | 3.21 |
|  | *XC_1358* | *pilT* | twitching motility protein PilT | 2.66 |
|  | *XC_1359* | *pilU* | twitching motility protein PilU | 4.04 |
|  | *XC_1619* | *oar* | Oar protein | 6.14 |
|  | *XC_1621* | *fimT* | pre-pilin like leader sequence | 7.77 |
|  | *XC_1622* | *pilV* | pre-pilin leader sequence | 11.58 |
|  | *XC_1624* | *pilX* | PilX protein | 7.46 |
|  | *XC_1626* | *pilE* | type IV pilus assembly protein PilE | 5.3 |
|  | *XC_3445* | *lgt* | prolipoprotein diacylglyceryl transferase | 3 |
|  | *XC_3629* |  | membrane protein WxcD | 2.75 |
|  | *XC_3640* | *uptC* | type II secretion system protein-like protein | 4.4 |
|  | *XC_3674* | *glmU* | UDP-N-acetylglucosamine pyrophosphorylase | 2.91 |
|  | *XC_4054* |  | SapC related protein | 4.61 |
|  | *XC_4077* |  | integral membrane protein | 2.99 |
|  | *XC_4094* |  | membrane protein | 2.82 |
|  | *XC_4099* | *plsC* | acyltransferase | 2.95 |
|  | *XC_4168* | *ndvB* | NdvB protein | 2.96 |
|  | *XC_4327* |  | Oar protein | 3.67 |
|  | *XC_3884* | *yiaA* | membrane protein | -2.63 |
| Cellular processes | *XC_1123* |  | alpha,alpha-trehalose-phosphate synthase | 3.79 |
|  | *XC_1409* | *cheB* | protein-glutamate methylesterase | 5.46 |
|  | *XC_1937* | *mcp* | methyl-accepting chemotaxis protein | 2.74 |
|  | *XC_2259* | *fliE* | flagellar hook-basal body complex protein FliE | -2.36 |
| Central intermediary metabolism | *XC_0165* | *pah* | phenylalanine hydroxylase | 4.86 |
|  | *XC_0303* |  | nucleoside hydrolase | 3.83 |
|  | *XC_0452* | *hmgA* | homogentisate 1,2-dioxygenase | 3.38 |
|  | *XC_0838* | *ilvA* | threonine dehydratase | 3.78 |
|  | *XC_0990* | *cysH* | 3'-phosphoadenosine 5'-phosphosulfate reductase | 7.66 |
|  | *XC_0991* | *cysI* | NADPH-sulfite reductase iron-sulfur protein | 6.61 |
|  | *XC_0992* | *cysJ* | NADPH-sulfite reductase flavoprotein subunit | 5.04 |
|  | *XC_0993* | *cysD* | ATP sulfurylase small subunit | 37.7 |
|  | *XC_0994* | *cysNC* | ATP sulfurylase | 5.37 |
|  | *XC_1002* | *malZ* | alpha-glucosidase | 2.54 |
|  | *XC_1003* |  | glycosyl hydrolase | 3.9 |
|  | *XC_1121* | *plc* | phospholipase C | 2.73 |
|  | *XC_1214* | *bga* | beta-galactosidase | 9.38 |
|  | *XC_1218* | *manB* | beta-mannosidase | 3.01 |
|  | *XC_1642* | *malZ* | alpha-glucosidase | 2.74 |
|  | *XC_2857* |  | protein U | 2.97 |
|  | *XC_2984* | *galA* | arabinogalactan endo-1,4-beta-galactosidase | 5.12 |
|  | *XC_2985* | *lacZ* | beta-galactosidase | 3.25 |
|  | *XC_2991* |  | beta-glucosidase | 5.68 |
|  | *XC_3135* | *gcvP* | glycine decarboxylase | 2.71 |
|  | *XC_3274* | *ppk* | polyphosphate kinase | 2.49 |
|  | *XC_3456* | *tauD* | taurine dioxygenase | 10.26 |
|  | *XC_3491* | *gal* | D-galactose 1-dehydrogenase | 3.13 |
|  | *XC_3719* | *dadA* | D-amino-acid dehydrogenase | 4.1 |
|  | *XC_4065* | *xynB* | beta-xylosidase | 4.56 |
|  | *XC_4158* |  | 2,4-diketo-3-deoxy-L-fuconate hydrolase | 3.34 |
|  | *XC_4191* | *xylA* | xylose isomerase | 2.96 |
|  | *XC_4198* | *uxuB* | fructuronate reductase | 2.7 |
|  | *XC_4205* | *gnl* | gluconolactonase precursor | 2.7 |
|  | *XC_4208* | *lacZ* | beta-galactosidase | 3.24 |
|  | *XC_0372* | *glpD* | glycerol-3-phosphate dehydrogenase | -4.92 |
|  | *XC_2458* | *gmuG* | mannan endo-1,4-beta-mannosidase | -2.75 |
| Energy and carbon metabolism | *XC_0098* | *fbp* | fructose-1,6-bisphosphatase | 2.78 |
|  | *XC_0247* | *aceB* | malate synthase | 4 |
|  | *XC_0279* | *dkgB* | oxidoreductase | 4.12 |
|  | *XC_0296* |  | oxidoreductase | 3.23 |
|  | *XC_0328* | *ssuE* | NADH-dependent FMN reductase | 7.57 |
|  | *XC_0595* |  | oxidoreductase | 3.17 |
|  | *XC_0779* |  | oxidoreductase | 6.54 |
|  | *XC_0890* |  | electron transfer protein azurin I | 3.61 |
|  | *XC_1197* |  | cytochrome P450 hydroxylase | 4.98 |
|  | *XC_1590* | *nuoB* | NADH-quinone oxidoreductase subunit B | 4.12 |
|  | *XC_1884* | *cydB* | cytochrome D ubiquinol oxidase | 8.15 |
|  | *XC_1885* | *cydA* | cytochrome D ubiquinol oxidase subunit I | 6.49 |
|  | *XC_3287* |  | phosphoglycerate mutase | 2.74 |
|  | *XC_3479* | *ppc* | phosphoenolpyruvate carboxylase | 2.56 |
|  | *XC_3614* | *fixA* | electron transfer flavoprotein beta subunit | 2.64 |
|  | *XC_3678* | *atpD* | ATP synthase beta chain | 4.54 |
|  | *XC_3679* | *atpG* | ATP synthase gamma chain | 4.77 |
|  | *XC_3680* | *atpA* | ATP synthase alpha chain | 3.33 |
|  | *XC_3683* | *atpE* | ATP synthase C chain | 6.64 |
|  | *XC_3684* | *atpB* | ATP synthase A chain | 4.01 |
|  | *XC_1300* | *cydA* | quinol oxidase Oxidative phosphorylation | -10.71 |
|  | *XC_1301* | *cydB* | quinol oxidase | -7.01 |
|  | *XC_1384* |  | alcohol dehydrogenase | -4.1 |
|  | *XC_3167* |  | oxidoreductase | -2.07 |
|  | *XC_3774* |  | Zn-dependent alcohol dehydrogenase | -2.33 |
|  | *XC_4155* |  | D-threo-aldose 1-dehydrogenase | -2.2 |
| Fatty acid and phospholipid metabolism | *XC_0014* | *clsA/B* | cardiolipin synthase | 2.7 |
|  | *XC_0214* | *gpsA* | glycerol-3-phosphate dehydrogenase | 2.89 |
|  | *XC_0229* | *oleD* | 2-alkyl-3-oxoalkanoate reductase | 2.84 |
|  | *XC_0238* |  | acyltransferase | 3.07 |
|  | *XC_4137* |  | phospholipase A1 | 2.93 |
|  | *XC_4324* | *glpQ* | glycerophosphoryl diester phosphodiesterase | 3.18 |
| Nucleotide metabolism | *XC_0304* | *add* | adenosine deaminase | 3.08 |
|  | *XC_0322* | *purU* | formyltetrahydrofolate deformylase | 3.74 |
|  | *XC_0670* |  | cytosine deaminase | 2.85 |
|  | *XC_4075* | *nrdB* | ribonucleoside-diphosphate reductase beta chain | 2.91 |
| Regulatory functions | *XC_0778* |  | transcriptional regulator | 3.4 |
|  | *XC_0816* |  | transcriptional regulator luxR family | 2.48 |
|  | *XC_0850* |  | response regulator | 3.67 |
|  | *XC_0891* | *luxR* | ATP-dependent transcriptional regulator | 4.12 |
|  | *XC_0916* |  | transcriptional regulator blaI family | 7.2 |
|  | *XC_1022* |  | regulatory protein bphR | 4.66 |
|  | *XC_1153* | *iciA* | LysR family transcriptional regulator | 2.77 |
|  | *XC_1437* |  | transcriptional regulator lysR family | 3.31 |
|  | *XC_2075* |  | transcriptional regulator | 2.59 |
|  | *XC_2723* |  | transcriptional regulator | 3.51 |
|  | *XC_3197* | *pilH* | two-component system response regulator PilH | 3.62 |
|  | *XC_3561* |  | transcriptional regulator | 3.14 |
|  | *XC_2973* |  | regulatory protein | -2.39 |
| Replication and DNA metabolism | *XC_0132* | *comEB* | deoxycytidylate deaminase | 7.29 |
|  | *XC_0476* | *hsdR* | type I restriction enzyme | 3.58 |
|  | *XC_0477* | *hsdS* | type I restriction enzyme | 3.65 |
|  | *XC_0480* | *hsdM* | type I site-specific deoxyribonuclease | 3.75 |
|  | *XC_0536* |  | helicase | 2.57 |
|  | *XC_0537* |  | ATP-dependent DNA helicase | 2.78 |
|  | *XC_1207* | *hsdM* | type I restriction enzyme M protein | 2.89 |
|  | *XC_1656* | *gumA* | integration host factor alpha chain | 25.73 |
|  | *XC_2785* |  | helicase | 3.86 |
|  | *XC_3035* | *mutS* | DNA mismatch repair protein | 4.15 |
|  | *XC_3180* | *hsdR* | type I restriction enzyme | 3.86 |
|  | *XC_3181* | *hsdS* | type I restriction enzyme | 3.26 |
|  | *XC_3183* |  | type I restriction-modification system, M subunit, putative | 5.42 |
|  | *XC_3184* | *mrr* | restriction system protein | 4.2 |
|  | *XC_3499* | *mutT* | 8-oxo-dGTP diphosphatase | 3.18 |
|  | *XC_3603* | *alkB* | DNA repair system specific for alkylated DNA | 4.96 |
|  | *XC_3735* | *udg* | uracil-DNA glycosylase | 4.31 |
|  | *XC_4038* |  | ATP-dependent helicase | 3.34 |
|  | *XC_4115* | *uvrD* | DNA helicase II | 2.95 |
|  | *XC_4135* | *xthA1* | exodeoxyribonuclease III | 4.29 |
|  | *XC_4234* | *hsdM* | type I restriction enzyme M protein, XmnI methyltransferase | 2.68 |
|  | *XC_1234* |  | histone H1 | -2.26 |
|  | *XC_2186* | *xthA* | exodeoxyribonuclease | -2.03 |
| Transport | *XC_0084* | *proP* | proline/betaine transporter | 6.64 |
|  | *XC_0167* |  | ferripyoverdine receptor | 4.33 |
|  | *XC_0218* |  | MFS transporter | 24.58 |
|  | *XC_0305* | *pbuG* | putative MFS transporter | 3.73 |
|  | *XC_0361* |  | MFS transporter | 8.21 |
|  | *XC_0409* | *bfeA* | TonB-dependent receptor | 2.66 |
|  | *XC_0435* |  | cation efflux system protein | 3.37 |
|  | *XC_0494* | *bfr* | bacterioferritin | 2.63 |
|  | *XC_0687* | *fepA* | TonB-dependent receptor | 4.53 |
|  | *XC_0759* | *btuB* | TonB-dependent receptor | 4.47 |
|  | *XC_0885* |  | iron complex outermembrane recepter protein | 4.41 |
|  | *XC_0910* | *modA* | molybdate-binding periplasmic protein | 2.8 |
|  | *XC_0999* |  | sodium/proton-dependent alanine carrier protein | 5.03 |
|  | *XC_1004* |  | TonB-dependent receptor | 2.81 |
|  | *XC_1079* |  | iron complex outermembrane recepter protein | 4.4 |
|  | *XC_1104* | *iucA* | iron transporter | 9.69 |
|  | *XC_1112* |  | ferric enterobactin receptor | 4.12 |
|  | *XC_1113* |  | ferric enterobactin receptor | 2.87 |
|  | *XC_1122* |  | TonB-dependent receptor | 3.68 |
|  | *XC_1154* | *lysE* | membrane transport protein | 2.9 |
|  | *XC_1241* | *btuB* | TonB-dependent receptor | 6.24 |
|  | *XC_1706* |  | amino acid permease | 4.36 |
|  | *XC_2844* | *brf* | bacterioferritin | 6.52 |
|  | *XC_3205* |  | TonB-dependent receptor | 4.13 |
|  | *XC_3209* | *fyuA* | TonB-dependent receptor | 3.82 |
|  | *XC_3457* |  | ABC transporter substrate binding protein | 7.19 |
|  | *XC_3458* | *nrtCD* | ABC transporter ATP-binding component | 7.21 |
|  | *XC_3459* |  | permease | 5.27 |
|  | *XC_3463* | *phuR* | outer membrane hemin receptor | 7.89 |
|  | *XC_4053* | *iroN* | TonB-dependent receptor | 4.06 |
|  | *XC_4079* | *mgtE* | magnesium transporter | 3.17 |
|  | *XC_4169* |  | cation symporter | 3.82 |
|  | *XC_4249* |  | iron complex outermembrane recepter protein | 3.07 |
|  | *XC_4325* |  | iron complex outermembrane recepter protein | 3.91 |
|  | *XC_0807* | *suc1* | sugar transporter | -3.64 |
|  | *XC_3454* |  | ABC transporter ATP-binding protein | -2.48 |
|  | *XC_4146* | *ppa* | solute:Na+ symporter | -2.29 |
| Translation | *XC_0094* | *tldD* | TldD protein | 3.6 |
|  | *XC_0096* | *tldD* | TldD protein | 4.18 |
|  | *XC_0302* | *gatA* | glu-tRNAGln amidotransferase A subunit | 3.23 |
|  | *XC_0602* |  | aminopeptidase N | 7.71 |
|  | *XC_1291* |  | endoproteinase Arg-C | 4.46 |
|  | *XC_3266* |  | peptidyl-prolyl cis-trans isomerase | 2.84 |
|  | *XC_3315* | *rplQ* | 50S ribosomal protein L17 | 3.27 |
|  | *XC_3317* | *rpsD* | 30S ribosomal protein s4 | 3.01 |
|  | *XC_3319* | *rpsM* | 30S ribosomal protein S13 | 3.27 |
|  | *XC_3339* | *rplD* | 50S ribosomal protein L4 | 3.01 |
|  | *XC_3340* | *rplC* | 50S ribosomal protein L3 | 3.81 |
|  | *XC_3344* | *rpsG* | 30S ribosomal protein S7 | 2.68 |
|  | *XC_3575* |  | serine protease | 2.9 |
|  | *XC_3986* |  | protease Do | 3.14 |
|  | *XC_4009* |  | peptidase | 2.91 |
|  | *XC_4051* |  | dipeptidyl peptidase | 2.66 |
|  | *XC_4122* | *rpmG* | 50S ribosomal protein L33 | 4.32 |
|  | *XC_4123* | *rpmB* | 50S ribosomal protein L28 | 3.05 |
|  | *XC_1350* | *pfpI* | protease | -2.11 |
|  | *XC_2964* | *trmA* | RNA methyltransferase | -2.14 |
| Transcription | *XC_0438* |  | ATP-dependent RNA helicase | 2.46 |
|  | *XC_0478* |  | anticodon nuclease | 4.71 |
|  | *XC_0960* | *rph* | ribonuclease PH | 3.2 |
|  | *XC_1193* | *rpoE* | RNA polymerase sigma-70 factor | 4.07 |
|  | *XC_1609* | *pnp* | polynucleotide phosphorylase | 2.67 |
|  | *XC_3316* | *rpoA* | RNA polymerase alpha subunit | 3.89 |
|  | *XC_3643* | *rhlE* | ATP-dependent RNA helicase | 2.76 |
| Signal transduction | *XC_1526* |  | response regulator | 3.07 |
|  | *XC_1528* |  | sensor histidine kinase | 3.77 |
|  | *XC_2129* |  | sensor histidine kinase | 2.73 |
|  | *XC_3067* |  | response regulator | 4.37 |
|  | *XC_3068* |  | sensor histidine kinase | 2.59 |
|  | *XC_3669* |  | response regulator | 2.59 |
|  | *XC_3800* |  | two-component system sensor protein | 2.42 |
|  | *XC_4167* |  | histidine kinase | 3.26 |
|  | *XC_2456* | *regB* | sensor histidine kinase | -2.02 |
|  | *XC_2457* | *regR* | response regulator | -3.05 |
| Mobile genetic elements | *XC_0133* | *IS1479* | IS1479 transposase | 2.87 |
|  | *XC_0545* | *IS1477* | IS1477 transposase ORFA | 4.42 |
|  | *XC_0546* | *IS1477* | IS1477 transposase ORFB | 3.52 |
|  | *XC_0858* | *IS1477* | IS1477 transposase | 3.85 |
|  | *XC_1018* |  | phage-related integrase | 2.91 |
|  | *XC_1032* | *ISxcc1* | ISxcc1 transposase | 5.48 |
|  | *XC_1054* |  | transposase | 6.31 |
|  | *XC_1643* | *IS1479* | IS1479 transposase | 3.96 |
|  | *XC_2012* | *IS1479* | IS1479 transposase | 2.55 |
|  | *XC_2292* | *IS1478* | IS1478 transposase | 3.51 |
|  | *XC_2437* |  | Escherichia coli plasmid ColIa (fragment) | 6.74 |
|  | *XC_2438* |  | plasmid-related protein | 4.21 |
|  | *XC_2625* | *IS1404* | IS1404 transposase | 206.74 |
|  | *XC_2626* |  | IS1404 transposase | 3.87 |
|  | *XC_2783* | *IS1477* | IS1477 transposase | 4.03 |
|  | *XC_2993* | *IS1479* | IS1479 transposase | 4.84 |
|  | *XC_3623* |  | ISxcc1 transposase | 4.07 |
|  | *XC_3624* | *ISxcC1* | ISxcC1 transposase | 4.55 |
|  | *XC_3672* | *IS1404* | IS1404 transposase | 5.48 |
|  | *XC_3908* | *IS1478* | IS1478 transposase | 3.06 |
|  | *XC_2417* |  | plasmid mobilization protein | -37.36 |
|  | *XC_2399* |  | integrase/recombinase | -2.12 |
| Pathogenicity and adaptation | *XC_0027* | *egl* | endoglucanase | 7.59 |
|  | *XC_0411* |  | isopenicillin N epimerase | 4.79 |
|  | *XC_0479* |  | virulence protein RhuM family | 3.07 |
|  | *XC_0616* |  | putative thioredoxin reductase | 3.95 |
|  | *XC_0626* | *cbhA* | 1,4-beta-cellobiosidase | 2.93 |
|  | *XC_0644* |  | sulfur deprivation response regulator | 2.85 |
|  | *XC_0705* | *pehA* | endo-polygalacturonase | 7.02 |
|  | *XC_0864* | *virB6* | type IV secretion system protein VirB6 | 2.89 |
|  | *XC_1005* |  | 1,4-beta-cellobiosidase | 7.1 |
|  | *XC_1027* | *virB6* | type IV secretion system protein VirB6 | 5.04 |
|  | *XC_1217* |  | glucan 1,4-beta-glucosidase | 6.18 |
|  | *XC_1432* |  | multidrug resistance protein | 4.23 |
|  | *XC_1442* |  | extracellular serine protease | 3.87 |
|  | *XC_1449* |  | extracellular serine protease | 10.57 |
|  | *XC_1450* |  | extracellular serine protease | 14.37 |
|  | *XC_1514* |  | extracellular protease | 4.22 |
|  | *XC_1625* | *pilY* | Tfp pilus adhesin | 5.62 |
|  | *XC_1634* | *virB* | type IV secretion system protein VirB10 | 3.24 |
|  | *XC_1635* | *virB11* | type IV secretion system protein VirB11 | 4.13 |
|  | *XC_1636* | *virB1* | type IV secretion system protein VirB1 | 2.91 |
|  | *XC_1639* | *virB4* | type IV secretion system protein VirB4 | 4.57 |
|  | *XC_1668* | *gumL* | GumL protein | 3.8 |
|  | *XC_1669* | *gumM* | beta-1,4-glucosyltransferase | 2.73 |
|  | *XC_2845* |  | penicillin amidase | 3.22 |
|  | *XC_2955* | *cspA* | cold shock protein | 6.14 |
|  | *XC_2994* | *xopP* | XopP protein | 3.62 |
|  | *XC_3001* | *hpa2* | lysozyme-related protein Hpa2 | 7.34 |
|  | *XC_3010* | *hrpB2* | type III secretion inner rod protein HrpB2 | 2.87 |
|  | *XC_3012* | *hrcU* | type III secretion protein HrcU | 2.68 |
|  | *XC_3016* | *hrcR* | type III secretion protein HrcR | 3.64 |
|  | *XC_3024* | *xopF1* | XopF1 protein | 3.55 |
|  | *XC_3025* | *hrpF* | type III secretion translocon protein HrpF | 3.1 |
|  | *XC_3177* | *xopQ* | XopQ protein | 3.89 |
|  | *XC_3182* |  | virulence protein | 5.7 |
|  | *XC_3379* | *prtA* | extracellular protease | 4.48 |
|  | *XC_3590* | *pel* | pectate lyase | 3.71 |
|  | *XC_3591* | *pel* | pectate lyase | 8.51 |
|  | *XC_3620* |  | sugar translocase | 4.2 |
|  | *XC_3632* |  | lipopolysaccharide transport system ATP-binding protein | 2.67 |
|  | *XC_3633* |  | membrane subunit of LPS efflux transporter | 3.92 |
|  | *XC_4057* |  | TonB-like protein | 4.72 |
|  | *XC_4073* | *trxA* | thioredoxin | 3.29 |
|  | *XC_4127* | *czcC* | cation efflux system protein | 4.52 |
|  | *XC_4200* |  | bleomycin resistance protein | 3.48 |
|  | *XC_4207* | *xynA* | endo-1,4-beta-xylanase A | 2.63 |
|  | *XC_4256* |  | fusaric acid resistance protein | 3.63 |
|  | *XC_0625* |  | extracellular endoglucanase precursor | -2.09 |
|  | *XC_3754* |  | putative manganese-containing catalase | -2.18 |
| Undefined category | *XC_0015* |  | radical SAM domain protein | 3.61 |
|  | *XC_0136* |  | RhsD protein | 3.18 |
|  | *XC_0271* | *zapE* | cell division protein | 3.16 |
|  | *XC_0341* | *attT* | putative acetyltransferase | 11.5 |
|  | *XC_0360* |  | transferase | 3.46 |
|  | *XC_0568* | *rutE* | nitroreductase | 6.37 |
|  | *XC_0671* | *elaA* | ElaA protein | 3.88 |
|  | *XC_0888* | *tdcF* | TdcF protein | 4.15 |
|  | *XC_0962* | *rdgB* | diphosphohydrolase | 2.79 |
|  | *XC_1001* | *pnbA* | carboxylesterase | 2.75 |
|  | *XC_1198* |  | sulfotransferase | 5.64 |
|  | *XC_2078* |  | YeeB-like protein | 2.65 |
|  | *XC_2079* |  | YeeC-like protein | 3.23 |
|  | *XC_2592* |  | RhsD protein | 2.9 |
|  | *XC_2843* | *tpmT* | thiopurine S-methyltransferase | 3.91 |
|  | *XC_4159* | *fucD* | L-fuconate dehydratase | 3.64 |
|  | *XC_3972* |  | hydrolase/peptidase | -2.53 |
| Hypothetical proteins | *XC_0016* |  | conserved hypothetical protein | 3.49 |
|  | *XC_0019* |  | conserved hypothetical protein | 2.79 |
|  | *XC_0059* |  | conserved hypothetical protein | 3.7 |
|  | *XC_0062* |  | conserved hypothetical protein | 7.98 |
|  | *XC_0064* |  | conserved hypothetical protein | 3.31 |
|  | *XC_0065* |  | conserved hypothetical protein | 2.56 |
|  | *XC_0066* |  | conserved hypothetical protein | 4.42 |
|  | *XC_0067* |  | hypothetical protein | 4.78 |
|  | *XC_0073* |  | conserved hypothetical protein | 3.72 |
|  | *XC_0074* |  | conserved hypothetical protein | 2.96 |
|  | *XC_0103* |  | conserved hypothetical protein | 3.04 |
|  | *XC_0119* |  | conserved hypothetical protein | 3.05 |
|  | *XC_0120* |  | conserved hypothetical protein | 6.3 |
|  | *XC_0128* |  | conserved hypothetical protein | 10.44 |
|  | *XC_0129* |  | conserved hypothetical protein | 10.8 |
|  | *XC_0130* |  | conserved hypothetical protein | 8.76 |
|  | *XC_0131* |  | conserved hypothetical protein | 9.13 |
|  | *XC_0148* |  | conserved hypothetical protein | 3.64 |
|  | *XC_0180* |  | conserved hypothetical protein | 7.4 |
|  | *XC_0211* |  | conserved hypothetical protein | 5.01 |
|  | *XC_0212* |  | conserved hypothetical protein | 2.7 |
|  | *XC_0220* |  | conserved hypothetical protein | 3.51 |
|  | *XC_0236* |  | conserved hypothetical protein | 2.4 |
|  | *XC_0270* |  | conserved hypothetical protein | 2.68 |
|  | *XC_0272* |  | conserved hypothetical protein | 4.23 |
|  | *XC_0297* |  | conserved hypothetical protein | 10.47 |
|  | *XC_0329* |  | conserved hypothetical protein | 26.43 |
|  | *XC_0331* |  | conserved hypothetical protein | 5 |
|  | *XC_0337* |  | conserved hypothetical protein | 4.53 |
|  | *XC_0338* |  | conserved hypothetical protein | 5.01 |
|  | *XC_0340* |  | conserved hypothetical protein | 12.76 |
|  | *XC_0342* |  | conserved hypothetical protein | 9.02 |
|  | *XC_0344* |  | conserved hypothetical protein | 5.7 |
|  | *XC_0346* |  | conserved hypothetical protein | 5.87 |
|  | *XC_0347* |  | conserved hypothetical protein | 14.29 |
|  | *XC_0349* |  | conserved hypothetical protein | 6.03 |
|  | *XC_0350* |  | conserved hypothetical protein | 10.08 |
|  | *XC_0351* |  | conserved hypothetical protein | 4.45 |
|  | *XC_0352* |  | conserved hypothetical protein | 9.09 |
|  | *XC_0353* |  | conserved hypothetical protein | 5.06 |
|  | *XC_0359* |  | conserved hypothetical protein | 6.25 |
|  | *XC_0369* |  | conserved hypothetical protein | 4.53 |
|  | *XC_0442* |  | conserved hypothetical protein | 3.88 |
|  | *XC_0482* |  | conserved hypothetical protein | 4.33 |
|  | *XC_0538* |  | conserved hypothetical protein | 5.18 |
|  | *XC_0539* |  | conserved hypothetical protein | 5.51 |
|  | *XC_0540* |  | conserved hypothetical protein | 3.63 |
|  | *XC_0543* |  | conserved hypothetical protein | 3.01 |
|  | *XC_0563* |  | conserved hypothetical protein | 3.48 |
|  | *XC_0564* |  | conserved hypothetical protein | 3.19 |
|  | *XC_0566* |  | conserved hypothetical protein | 2.98 |
|  | *XC_0596* |  | conserved hypothetical protein | 3.34 |
|  | *XC_0604* |  | conserved hypothetical protein | 5.26 |
|  | *XC_0605* |  | conserved hypothetical protein | 5.49 |
|  | *XC_0607* |  | conserved hypothetical protein | 6.21 |
|  | *XC_0608* |  | conserved hypothetical protein | 4.17 |
|  | *XC_0617* |  | conserved hypothetical protein | 7.47 |
|  | *XC_0619* |  | conserved hypothetical protein | 6.55 |
|  | *XC_0622* |  | conserved hypothetical protein | 3.31 |
|  | *XC_0653* |  | conserved hypothetical protein | 10.06 |
|  | *XC_0710* |  | conserved hypothetical protein | 3.94 |
|  | *XC_0734* |  | conserved hypothetical protein | 13.66 |
|  | *XC_0791* |  | conserved hypothetical protein | 6.12 |
|  | *XC_0792* |  | conserved hypothetical protein | 4.72 |
|  | *XC_0793* |  | conserved hypothetical protein | 5.57 |
|  | *XC_0798* |  | conserved hypothetical protein | 4.46 |
|  | *XC_0855* |  | conserved hypothetical protein | 3.18 |
|  | *XC_0857* |  | conserved hypothetical protein | 3.97 |
|  | *XC_0860* |  | conserved hypothetical protein | 5.08 |
|  | *XC_0861* |  | conserved hypothetical protein | 10.51 |
|  | *XC_0870* |  | conserved hypothetical protein | 3.67 |
|  | *XC_0914* |  | conserved hypothetical protein | 5.15 |
|  | *XC_0917* |  | conserved hypothetical protein | 3.51 |
|  | *XC_0961* |  | conserved hypothetical protein | 3.39 |
|  | *XC_0967* |  | conserved hypothetical protein | 11.68 |
|  | *XC_0973* |  | conserved hypothetical protein | 5.8 |
|  | *XC_0974* |  | conserved hypothetical protein | 3.67 |
|  | *XC_0986* |  | conserved hypothetical protein | 2.4 |
|  | *XC_1019* |  | conserved hypothetical protein | 5.95 |
|  | *XC_1020* |  | conserved hypothetical protein | 2.97 |
|  | *XC_1023* |  | conserved hypothetical protein | 5.88 |
|  | *XC_1028* |  | conserved hypothetical protein | 3.14 |
|  | *XC_1029* |  | conserved hypothetical protein | 4.98 |
|  | *XC_1030* |  | conserved hypothetical protein | 6.06 |
|  | *XC_1041* |  | conserved hypothetical protein | 3.02 |
|  | *XC_1042* |  | conserved hypothetical protein | 4.27 |
|  | *XC_1089* |  | conserved hypothetical protein | 3.23 |
|  | *XC_1106* |  | conserved hypothetical protein | 6.96 |
|  | *XC_1107* |  | conserved hypothetical protein | 10.17 |
|  | *XC_1203* |  | conserved hypothetical protein | 3.17 |
|  | *XC_1208* |  | conserved hypothetical protein | 3.11 |
|  | *XC_1215* |  | conserved hypothetical protein | 6.28 |
|  | *XC_1216* |  | conserved hypothetical protein | 6.43 |
|  | *XC_1230* |  | conserved hypothetical protein | 3.2 |
|  | *XC_1239* |  | conserved hypothetical protein | 2.75 |
|  | *XC_1271* |  | conserved hypothetical protein | 2.38 |
|  | *XC_1288* |  | conserved hypothetical protein | 6.18 |
|  | *XC_1336* |  | conserved hypothetical protein | 3.6 |
|  | *XC_1448* |  | conserved hypothetical protein | 6.25 |
|  | *XC_1534* |  | conserved hypothetical protein | 2.56 |
|  | *XC_1558* |  | conserved hypothetical protein | 4.03 |
|  | *XC_1559* |  | conserved hypothetical protein | 3.73 |
|  | *XC_1585* |  | conserved hypothetical protein | 4.47 |
|  | *XC_1620* |  | conserved hypothetical protein | 4.24 |
|  | *XC_1623* |  | conserved hypothetical protein | 6.27 |
|  | *XC_1718* |  | conserved hypothetical protein | 4.63 |
|  | *XC_1719* |  | hypothetical protein | 2.5 |
|  | *XC_1765* |  | conserved hypothetical protein | 11.5 |
|  | *XC_1915* |  | conserved hypothetical protein | 2.02 |
|  | *XC_1921* |  | conserved hypothetical protein | 4.9 |
|  | *XC_2123* |  | hypothetical protein | 12.53 |
|  | *XC_2288* |  | conserved hypothetical protein | 2.6 |
|  | *XC_2289* |  | conserved hypothetical protein | 2.72 |
|  | *XC_2291* |  | conserved hypothetical protein | 5.04 |
|  | *XC_2577* |  | conserved hypothetical protein | 2.99 |
|  | *XC_2633* |  | conserved hypothetical protein | 3.17 |
|  | *XC_2847* |  | conserved hypothetical protein | 2.81 |
|  | *XC_2989* |  | conserved hypothetical protein | 5 |
|  | *XC_2990* |  | conserved hypothetical protein | 5.43 |
|  | *XC_2992* |  | conserved hypothetical protein | 10.01 |
|  | *XC_2999* |  | conserved hypothetical protein | 6.06 |
|  | *XC_3000* |  | conserved hypothetical protein | 3.83 |
|  | *XC_3031* |  | conserved hypothetical protein | 5.37 |
|  | *XC_3033* |  | conserved hypothetical protein | 4.1 |
|  | *XC_3113* |  | conserved hypothetical protein | 8.16 |
|  | *XC_3168* |  | conserved hypothetical protein | 4.02 |
|  | *XC_3189* |  | conserved hypothetical protein | 4.11 |
|  | *XC_3190* |  | conserved hypothetical protein | 3.37 |
|  | *XC_3219* |  | conserved hypothetical protein | 6.22 |
|  | *XC_3220* |  | conserved hypothetical protein | 3.67 |
|  | *XC_3286* |  | conserved hypothetical protein | 5.43 |
|  | *XC_3305* |  | conserved hypothetical protein | 4.5 |
|  | *XC_3417* |  | conserved hypothetical protein | 2.61 |
|  | *XC_3461* |  | conserved hypothetical protein | 5.98 |
|  | *XC_3462* |  | conserved hypothetical protein | 6.53 |
|  | *XC_3540* |  | conserved hypothetical protein | 4.44 |
|  | *XC_3555* |  | conserved hypothetical protein | 3.7 |
|  | *XC_3595* |  | conserved hypothetical protein | 6.28 |
|  | *XC_3621* |  | conserved hypothetical protein | 2.76 |
|  | *XC_3645* |  | conserved hypothetical protein | 3.11 |
|  | *XC_3728* |  | conserved hypothetical protein | 2.82 |
|  | *XC_3736* |  | conserved hypothetical protein | 2.87 |
|  | *XC_3789* |  | conserved hypothetical protein | 3.02 |
|  | *XC_3798* |  | conserved hypothetical protein | 2.68 |
|  | *XC_3813* |  | conserved hypothetical protein | 4.81 |
|  | *XC_3820* |  | conserved hypothetical protein | 3.58 |
|  | *XC_3851* |  | conserved hypothetical protein | 4.25 |
|  | *XC_3883* |  | conserved hypothetical protein | 2.43 |
|  | *XC_3909* |  | conserved hypothetical protein | 6.15 |
|  | *XC_3910* |  | conserved hypothetical protein | 5.43 |
|  | *XC_3911* |  | conserved hypothetical protein | 10.69 |
|  | *XC_3912* |  | conserved hypothetical protein | 9.02 |
|  | *XC_3913* |  | conserved hypothetical protein | 3.77 |
|  | *XC_3939* |  | conserved hypothetical protein | 3.37 |
|  | *XC_3969* |  | conserved hypothetical protein | 5.26 |
|  | *XC_3970* |  | conserved hypothetical protein | 2.7 |
|  | *XC_3996* |  | conserved hypothetical protein | 2.56 |
|  | *XC_4074* |  | conserved hypothetical protein | 3.72 |
|  | *XC_4112* |  | conserved hypothetical protein | 7.39 |
|  | *XC_4113* |  | conserved hypothetical protein | 15 |
|  | *XC_4166* |  | conserved hypothetical protein | 7.43 |
|  | *XC_4188* |  | conserved hypothetical protein | 2.84 |
|  | *XC_4189* |  | conserved hypothetical protein | 3.8 |
|  | *XC_4190* |  | conserved hypothetical protein | 2.87 |
|  | *XC_4199* |  | conserved hypothetical protein | 6.04 |
|  | *XC_4204* |  | conserved hypothetical protein | 2.6 |
|  | *XC_4217* |  | conserved hypothetical protein | 5.03 |
|  | *XC_4250* |  | conserved hypothetical protein | 3.23 |
|  | *XC_4255* |  | conserved hypothetical protein | 7.02 |
|  | *XC_4275* |  | hypothetical protein | 3.01 |
|  | *XC_4304* |  | conserved hypothetical protein | 4.16 |
|  | *XC_0343* |  | conserved hypothetical protein | -6.05 |
|  | *XC_0934* |  | conserved hypothetical protein | -2.12 |
|  | *XC_1302* |  | conserved hypothetical protein | -10.05 |
|  | *XC_1400* |  | conserved hypothetical protein | -2.29 |
|  | *XC_1687* |  | conserved hypothetical protein | -2.49 |
|  | *XC_1910* |  | conserved hypothetical protein | -2.52 |
|  | *XC_1916* |  | conserved hypothetical protein | -2.83 |
|  | *XC_2142* |  | conserved hypothetical protein | -2.19 |
|  | *XC_2414* |  | hypothetical protein | -4.07 |
|  | *XC_2415* |  | conserved hypothetical protein | -22.75 |
|  | *XC_2416* |  | hypothetical protein | -103.15 |
|  | *XC_2545* |  | conserved hypothetical protein | -2.18 |
|  | *XC_2646* |  | conserved hypothetical protein | -2.05 |
|  | *XC_2647* |  | conserved hypothetical protein | -2.08 |
|  | *XC_2759* |  | hypothetical protein | -2.87 |
|  | *XC_2921* |  | conserved hypothetical protein | -2.11 |
|  | *XC_2933* |  | conserved hypothetical protein | -3.2 |
|  | *XC_3446* |  | conserved hypothetical protein | -2.43 |
|  | *XC_3711* |  | conserved hypothetical protein | -2.65 |
|  | *XC_3753* |  | conserved hypothetical protein | -3.55 |
|  | *XC_3755* |  | conserved hypothetical protein | -3.24 |
|  | *XC_3756* |  | conserved hypothetical protein | -3.03 |
|  | *XC_3773* |  | conserved hypothetical protein | -3 |
|  | *XC_3855* |  | conserved hypothetical protein | -2.85 |
|  | *XC_3882* |  | conserved hypothetical protein | -2.68 |
|  | *XC_3971* |  | conserved hypothetical protein | -3.15 |
|  | *XC_4147* |  | conserved hypothetical protein | -2.05 |

Note: False discovery rate (FDR) =0.05 and absolute value of log_2_FC(log_2_foldchange) =1 (equivalent to a fold change of 2) were used as the cut off values.“+” represents gene up-regulated, and “-”represents gene down-regulated.
