# Supplementary material for: A HU‐like protein is required for full virulence in Xanthomonas campestris pv. campestris
Source: Mol Plant Pathol. 2021 Aug 23;22(12):1574–86. doi: 10.1111/mpp.13128 (PMC8578834; doi:10.1111/mpp.13128)
Supplement: Supplementary file 11 — TABLE S3 Confirmation of RNA‐seq gene expression data by semiquantitative reverse‐transcription PCR [file MPP-22-1574-s004.docx]

Table S3. Confirmation of RNA‐Seq gene expression data by semi‐quantitative RT‐PCR

| ID | Gene | Annotation | Expresstion level | Semi RT-PCR wt/∆hlp |
| --- | --- | --- | --- | --- |
| *XC_0167* |  | ferripyoverdine receptor | 4.33↑ | 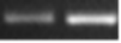 |
| *XC_0576* | *adcG* | phosphoribosyl-dephospho-CoA transferase | -2.83↓ | 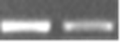 |
| *XC_0970* | *ompW* | outer membrane protein | 9.26↑ | 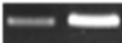 |
| *XC_0992* | *cysJ* | NADPH-sulfite reductase flavoprotein subunit | 5.04↑ | 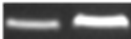 |
| *XC_1004* |  | TonB-dependent receptor | 2.81↑ | 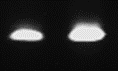 |
| *XC_1187* | *pilL* | PilL protein,pilin biosynthetic protein | 4.74↑ | 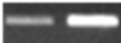 |
| *XC_1214* | *bga* | beta-galactosidase | 9.38↑ | 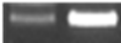 |
| *XC_1350* | *pfpI* | protease | -2.11↓ | 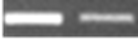 |
| *XC_1358* | *pilT* | twitching motility protein PilT | 2.66↑ | 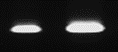 |
| *XC_1450* |  | extracellular serine protease | 14.37↑ | 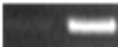 |
| *XC_1621* | *fimT* | pre-pilin like leader sequence | 7.77↑ | 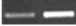 |
| *XC_1626* | *pilE* | type IV pilus assembly protein PilE | 5.3↑ | 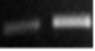 |
| *XC_2259* | *fliE* | flagellar hook-basal body complex protein FliE | -2.36↓ | 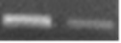 |
| *XC_2415* |  | conserved hypothetical protein | -22.75↓ | 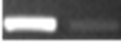 |
| *XC_2633* |  | conserved hypothetical protein | 3.17↑ | 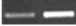 |
| *XC_2723* |  | transcriptional regulator | 3.51↑ | 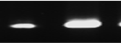 |
| *XC_2857* |  | protein U | 2.97↑ | 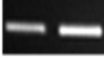 |
| *XC_3016* | *hrcR* | type III secretion protein HrcR | 3.64↑ | 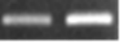 |
| *XC_3025* | *hrpF* | type III secretion translocon protein HrpF | 3.1↑ | 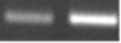 |
| *XC_3456* | *tauD* | taurine dioxygenase | 10.26↑ | 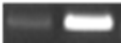 |
| *XC_3461* |  | conserved hypothetical protein | 5.98↑ | 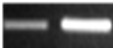 |
| *XC_3463* | *phuR* | outer membrane hemin receptor | 7.89↑ | 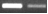 |
| *XC_3575* |  | serine protease | 2.9↑ | 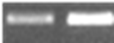 |
| *XC_3591* | *pel* | pectate lyase | 8.51↑ | 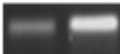 |
| *XC_3754* |  | putative manganese-containing catalase | -2.18↓ | 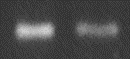 |
| *16S* |  |  |  | 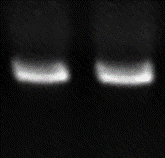 |

Note: RNA samples were processed using the same treatments as for RNA-Seq, and cDNA fragments were obtained by using a cDNA Synthesis kit (Invitrogen, Waltham, MA, USA). The 16S rRNA gene of Xcc 8004 was used as the internal control to verify the absence of significant variation at cDNA level in the samples. In this study, false discovery rate (FDR) ≤0.05 and absolute value of log2 fold change ≥1 were used as the cut off values. The acquired results were accordant to the transcriptome data.↑: up-regulated; ↓: down-regulated.
