# Supplementary material for: A HU‐like protein is required for full virulence in Xanthomonas campestris pv. campestris
Source: Mol Plant Pathol. 2021 Aug 23;22(12):1574–86. doi: 10.1111/mpp.13128 (PMC8578834; doi:10.1111/mpp.13128)
Supplement: Supplementary file 12 — TABLE S4 Genes coregulated by both Hlp and Flp [file MPP-22-1574-s008.docx]

**Table S4.** Genes regulated by both Hlp and Flp

| **Function Category** | **Gene ID** | **Name** | **Predicted product** | **Expression fold change ∆*hlp*** | **Expression fold change ∆*flp* (From Leng *et al*., 2019)** |
| --- | --- | --- | --- | --- | --- |
| **Amino acid biosynthesis** | *XC_0330* | *metE* | 5-methyltetrahydropteroyltriglutamate-homocysteine methyltransferase | 28.27 | -7.73 |
|  | *XC_1569* | *Asd* | aspartate semialdehyde dehydrogenase | 2.89 | 2.27 |
| **Cell envelope and cell structure** | *XC_1184* | *pilH* | two-component system response regulator PilH | 6.09 | 3.07 |
|  | *XC_1187* | *pilL* | PilL protein, pilin biosynthetic protein | 4.74 | 2.7 |
|  | *XC_1219* |  | beta-hexosaminidase | 3.21 | 2.08 |
|  | *XC_1358* | *pilT* | twitching motility protein PilT | 2.66 | 2.35 |
|  | *XC_1359* | *pilU* | twitching motility protein PilU | 4.04 | 2.01 |
|  | *XC_1619* | *oar* | Oar protein | 6.14 | 2.36 |
| **Cellular processes** | *XC_1123* |  | alpha,alpha-trehalose-phosphate synthase | 3.79 | 2.53 |
| **Central intermediary metabolism** | *XC_0452* | *hmgA* | homogentisate 1,2-dioxygenase | 3.38 | 2.35 |
|  | *XC_0990* | *cysH* | 3'-phosphoadenosine 5'-phosphosulfate reductase | 7.66 | 5.24 |
|  | *XC_0991* | *cysI* | NADPH-sulfite reductase iron-sulfur protein | 6.61 | 3.9 |
|  | *XC_0993* | *cysD* | ATP sulfurylase small subunit | 37.7 | 3.65 |
|  | *XC_1002* | *malZ* | alpha-glucosidase | 2.54 | 2.13 |
|  | *XC_1218* | *manB* | beta-mannosidase | 3.01 | 2.22 |
|  | *XC_1642* | *malZ* | alpha-glucosidase | 2.74 | 3.05 |
|  | *XC_3456* | *tauD* | taurine dioxygenase | 10.26 | 4.4 |
|  | *XC_4191* | *xylA* | xylose isomerase | 2.96 | 3.09 |
| **Energy and carbon metabolism** | *XC_0247* | *aceB* | malate synthase | 4.0 | 4.22 |
|  | *XC_0328* | *ssuE* | NADH-dependent FMN reductase | 7.57 | -2.04 |
|  | *XC_3167* |  | oxidoreductase | -2.07 | -2.55 |
| **Regulatory functions** | *XC_2723* |  | transcriptional regulator | 3.51 | 2.38 |
|  | *XC_2973* |  | regulatory protein | -2.39 | -5.94 |
| **Replication and DNA metabolism** | *XC_2785* |  | helicase | 3.86 | 2.37 |
| **Transport** | *XC_0218* |  | MFS transporter | 24.58 | -2.73 |
|  | *XC_1004* |  | TonB-dependent receptor | 2.81 | 4.73 |
|  | *XC_1104* | *iucA* | iron transporter | 9.69 | 3.67 |
|  | *XC_3458* | *nrtCD* | ABC transporter ATP-binding component | 7.21 | 4.07 |
|  | *XC_3459* |  | Permease | 5.27 | 2.09 |
|  | *XC_3463* | *phuR* | outer membrane hemin receptor | 7.89 | 2.37 |
|  | *XC_4146* | *ppa* | solute:Na^+^ symporter | -2.29 | 4.52 |
| **Translation** | *XC_3986* |  | protease Do | 3.14 | -2.23 |
| **Mobile genetic** | *XC_1643* | *IS1479* | IS1479 transposase | 3.96 | 2.65 |
| **Pathogenicity and adaptation** | *XC_0705* | *pehA* | endo-polygalacturonase | 7.02 | -2.12 |
|  | *XC_1005* |  | 1,4-beta-cellobiosidase | 7.1 | 4.9 |
|  | *XC_1027* | *virB6* | type IV secretion system protein VirB6 | 5.04 | 2.93 |
|  | *XC_1432* |  | multidrug resistance protein | 4.23 | 3.37 |
|  | *XC_1450* |  | extracellular serine protease | 14.37 | -3.4 |
|  | *XC_3010* | *hrpB2* | type III secretion inner rod protein HrpB2 | 2.87 | -4.33 |
|  | *XC_3012* | *hrcU* | type III secretion protein HrcU | 2.68 | -4.26 |
|  | *XC_3016* | *hrcR* | type III secretion protein HrcR | 3.64 | -5.79 |
|  | *XC_3025* | *hrpF* | type III secretion translocon protein HrpF | 3.1 | -3.54 |
|  | *XC_3590* | *pel* | pectate lyase | 3.71 | 16.1 |
|  | *XC_3591* | *pel* | pectate lyase | 8.51 | 14.61 |
|  | *XC_4200* |  | bleomycin resistance protein | 3.48 | 2.05 |
| **Hypothetical proteins** | *XC_0792* |  | conserved hypothetical protein | 4.72 | 2.18 |
|  | *XC_0793* |  | conserved hypothetical protein | 5.57 | 3.26 |
|  | *XC_1023* |  | conserved hypothetical protein | 5.88 | 3.18 |
|  | *XC_1107* |  | conserved hypothetical protein | 10.17 | 2.21 |
|  | *XC_1215* |  | conserved hypothetical protein | 6.28 | 2.33 |
|  | *XC_1559* |  | conserved hypothetical protein | 3.73 | 2.08 |
|  | *XC_1765* |  | conserved hypothetical protein | 11.5 | -2.09 |
|  | *XC_2123* |  | hypothetical protein | 12.53 | 10.16 |
|  | *XC_3305* |  | conserved hypothetical protein | 4.5 | -2.98 |
|  | *XC_3970* |  | conserved hypothetical protein | 2.7 | 2.04 |

Note: “+” represents gene up-regulated, and “-”represents gene down-regulated.

**Leng, M., Lu, Z.J., Qin, Z.S., Qi, Y.H., Lu, G.T. and Tang, J.L.** (2019) Flp, a Fis-like protein, contributes to the regulation of type III secretion and virulence processes in the phytopathogen *Xanthomonas campestris* pv. *campestris*. *Molecular plant pathology*, 20, 20, 1119-1133.
