# Supplementary material for: A HU‐like protein is required for full virulence in Xanthomonas campestris pv. campestris
Source: Mol Plant Pathol. 2021 Aug 23;22(12):1574–86. doi: 10.1111/mpp.13128 (PMC8578834; doi:10.1111/mpp.13128)
Supplement: Supplementary file 13 — TABLE S5 Primers used in this study [file MPP-22-1574-s002.docx]

**Table S5.** Primers used in this study^§^

| Primers | Nucleotide sequence (5′→3′) | The amplified fragment or the utilization |
| --- | --- | --- |
| L*flp*-F  L*flp*-R | ACAGTTGAATTCTGCAGCTGTCGCTGTCCGATGCCGA  ACAGTTGGATCCTGCGTTCAAAGGAGGTTCCCAGTGT | 333-bp DNA sequence upstream of *flp*, used for construction of *flp* deletion mutant. |
| R*flp*-F  R*flp*-R | ACAGTTGGATCCTATGGGCTGACGTAGGTTTGGGAATC  ACAGTTAAGCTTTCAGGCGACGGTTGGGACGCGCTCG | 339-bp DNA sequence downstream of *flp*, used for construction of *flp* deletion mutant. |
| C*flp*-F  C*flp*-R | ACAGTTGGATCCTTGAACGCAGCCCCCTCTCGTCCTG  ACAGTTAAGCTTCTACGTCAGCCCATACTCTT | DNA fragment of 270-bp *flp* coding sequence, used for complementation, overproduction and site-directed mutagenesis of *flp*. |
| 1046 ORF-F  1046 ORF-R | GGGGGATCCATGCGCCAAGGTTCGCCCGC B/E  GGGAAGCTTTCAGCCCGGATACAACGGAA | DNA fragment of 390-bp *XC_1046* coding sequence, used for cross-complementation. |
| 1234 ORF-F  1234 ORF-R | ACAGTTGGATCCATGGCCACCACTAAAAAAGC  ACAGTTAAGCTTTCAGATCAGCGGTGCCGGGG | DNA fragment of 468-bp *XC_1234* coding sequence, used for cross-complementation. |
| 1355 ORF-F  1355 ORF-R | GGGGGATCCATGGCAAAAACCGCCGCTAA  GGGAAGCTTTTACAGCGCGGCGTCCTTCA | DNA fragment of 420-bp *hlp* coding sequence, used for complementation. |
| 1656 ORF-F  1656 ORF-R | ACAGTTCTGCAGATGGCATTGACGAAAGCGGA  ACAGTTAAGCTTTTACTGCCCGGATCCAGCAT | DNA fragment of 297-bp *XC_1656* coding sequence, used for cross-complementation. |
| 1806 ORF-F  1806 ORF-R | GGGGGATCCGTGAGCAATACTTCTTCGAA  GGGAAGCTTTTACTCGCGAATCAGCGGGA | DNA fragment of 402-bp *XC_1806* coding sequence, used for cross-complementation. |
| 1860 ORF-F  1860 ORF-R | GGGGGATCCGTGGCTGCTAAAAAAACTGC  GGGAAGCTTTCAGCACAGCCAGGCATTGG | DNA fragment of 258-bp *XC_1860* coding sequence, used for cross-complementation. |
| 1925 ORF-F  1925 ORF-R | ACAGTTGGATCCATGACCAAGTCCGAATTGATCG  ACAGTTAAGCTTTCAGTCCGCTGCATCCACCA | DNA fragment of 309-bp *XC_1925* coding sequence, used for cross-complementation. |
| 3262 ORF-F  3262 ORF-R | GGGGGATCCATGAATAAAACCGAATTGATC  GGGAAGCTTTTAGTTTACTGCATCCTTCA | DNA fragment of 270-bp *XC_3262* coding sequence, used for cross-complementation. |
| 3597 ORF-F  3597 ORF-R | GGGGGATCCATGTCGATCGATCTCACCGG  GGGAAGCTTTCACGCAGCCTTGGCTTTCT | DNA fragment of 465-bp *XC_3597* coding sequence, used for cross-complementation. |
| 3985 ORF-F  3985 ORF-R | GGGGGATCCATGAGCAATGGCAATGGTGTGAC  ACAGTTAAGCTTTCAGCGCGTGCCGCGCGGAA | DNA fragment of 777-bp *XC_3985* coding sequence, used for cross-complementation. |
| 4203 ORF-F  4203 ORF-R | ACAGTTGGATCCGTGCGGAAGGGCGCCACCAAGAAG  ACAGTTAAGCTTCTACTCCGCGGCTTTCCTGG | DNA fragment of 249-bp *XC_4203* coding sequence, used for cross-complementation. |
| 1355LF  1355LR | CGGGATCCCTTCATCGAATGCGCTTCGT  CGTCTAGAGAGTTATGTGTTCCGTATTC | 439-bp DNA sequence upstream of *hlp*, used for construction of *flp* deletion mutant. |
| 1355RF  1355RR | CGTCTAGATAATCGCGCTGCATCCGGTT  CGAAGCTTTCTACCGGGATGTGCAACTG | 340-bp DNA sequence downstream of *hlp*, used for construction of *hlp* deletion mutant. |
| 0167-F  0167-R | CGAGTCGCTTTTCAGCATCG  TGACCGTGGCAGAGGGAATG | 248-bp DNA fragment spans nucleotides 270 to 517 bp of the *XC_0167*, used for RT-PCR. |
| 0576-F  0576-R | CTGGTTTGCAGCGGGGTTTC  ACGCAAAGCTGCCGAAGACG | 275-bp DNA fragment spans nucleotides 87 to 361 bp of the *XC_0576*, used for RT -PCR. |
| 0970-F  0970-R | CCAATCCAAGGGTGACTGGT  TGTTGATGTCGTGCTCGAAT | 200-bp DNA fragment spans nucleotides 69 to 268 bp of the *XC_0970*, used for RT-PCR. |
| 0992-F  0992-R | AGCGTCTGGTCGACGGTCTGGATGC  CATTGCCGGTCTGGCTGCCGTACAA | 177-bp DNA fragment spans nucleotides 65 to 241 bp of the *XC_0992*, used for RT-PCR. |
| 1004-F  1004-R | TTTCGGCTGAAGACATCGGC  TCGACGAAATCCGGCTGTGC | 226-bp DNA fragment spans nucleotides 212 to 437 bp of the *XC_1004*, used for RT -PCR. |
| 1187-F  1187-R | GGAGCTGGATGAAACGTTGC  CAGTTCCAGTTCTTCGGCCA | 172-bp DNA fragment spans nucleotides 105 to 276 bp of the *XC_1187*, used for RT-PCR. |
| 1214-F  1214-R | ACGGCAAGCCGTACCAAGTG  ATCGAATTGCCCCTGCTGCG | 161-bp DNA fragment spans nucleotides 125 to 285 bp of the *XC_1214*, used for RT-PCR. |
| 1350-F  1350-R | TCGCGACCAGTGGCTTCGAA  TTGATCACACCGCCTGGCAG | 217-bp DNA fragment spans nucleotides 38 to 254 bp of the *XC_1350*, used for RT-PCR. |
| 1358-F  1358-R | AAGGCATCGGACTTGCACCT  GGAATTCCTCGTAATCGCGG | 160-bp DNA fragment spans nucleotides 40 to 199 bp of the *XC_1358*, used for RT-PCR and qRT-PCR. |
| 1450-F  1450-R | GATACCTCGCGCTGTTGA  CTACATCCATGTCGACGC | 195-bp DNA fragment spans nucleotides 20 to 214 bp of the *XC_1450*, used for RT-PCR. |
| 1621-F  1621-R | CGGGTTCACGCTGGTTGAGT  GCCGTTACTGCTTCCACAGA | 202-bp DNA fragment spans nucleotides 21 to 222 bp of the *XC_1621*, used for RT-PCR. |
| 1626-F  1626-R | GCGATCTTGTCTGCCATTGC  GGGTGGTTTGAAGAGCGAGC | 211-bp DNA fragment spans nucleotides 34 to 244 bp of the *XC_1626*, used for RT-PCR. |
| 2259-F  2259-R | GATGCTGCGCGCGGAAATC  TTTCGAACGCCTTGGCCAGC | 154-bp DNA fragment spans nucleotides 31 to 184 bp of the *XC_2259*, used for RT-PCR. |
| 2415-F  2415-R | GCCATCATCGGCACGTTGATCG  CGTCACAGGTGTAGCTGGGCGC | 184-bp DNA fragment spans nucleotides 19 to 202 bp of the *XC_2415*, used for RT-PCR. |
| 2633-F  2633-R | TGAAACACGCAAGGATCGG  TTCTTGCTCACGGGGATGG | 265-bp DNA fragment spans nucleotides 2 to 266 bp of the *XC_2633*, used for RT-PCR. |
| 2723-F  2723-F | TGGATCTGGAAGACTGGTCG  CGTCAAACCGGTAGTAGGCC | 219-bp DNA fragment spans nucleotides 59 to 277 bp of the *XC_2723*, used for RT-PCR. |
| 2857-F  2857-R | CATCGCGACAGCAGCAGACA  TGGTCACATCGTTGGCCGTC | 230-bp DNA fragment spans nucleotides 120 to 349 bp of the *XC_2857*used for RT-PCR. |
| 3010-F  3010-R | GCGCTCATTCCTCCTGTTG  TTCCTCAGCCGCAATTTCC | 282-bp DNA fragment spans nucleotides 4 to 285 bp of the *XC_3010* (*hrpB2*), used for qRT-PCR. |
| 3012-F  3012-R | GGCAGCTATTTTGTCGAACATC  CTGCACAAAGGTGCCGATCAAT | 183-bp DNA fragment spans nucleotides 136 to 318 bp of the *XC_3012* (*hrcU*), used for qRT-PCR. |
| 3016-F  3016-R | CCTGATGTCGGCTCGCTG  CGCGGGTGTGCTTGAGCA | 319-bp DNA fragment spans nucleotides 10 to 328 bp of the *XC_3016* (*hrcR*), used for RT-PCR and qRT-PCR. |
| 3025-F  3025-R | AGCCGATTGAGAAACGGACCTCCT  GCGGCGTCTTGTCTTTGTGCTGATT | 183-bp DNA fragment spans nucleotides 257 to 439 bp of the *XC_3025* (*hrpF*), used for RT–PCR and qRT-PCR. |
| 3177-F  3177-R | CAAGCCATGGCCTTCGACCAGAT  CATCAACCGTTTTGCCTTCTCCG | 198-bp DNA fragment spans nucleotides 1111 to 1308 bp of the XC_3177 (*xopQ*), used for qRT-PCR. |
| 3456-F  3456-R | ACCACGTGCTGGTGTTCCGCGATCA  CAGTAATGGCCGGCATCACCGAGCC | 190-bp DNA fragment spans nucleotides 170 to 359 bp of the *XC_3456*, used for RT-PCR. |
| 3461-F  3461-R | GCGAGTACGCCGTGGACTTT  TCATAGGTCCAGTCCTGCCG | 202-bp DNA fragment spans nucleotides 119 to 320 bp of the *XC_3461* used for RT-PCR. |
| 3463-F  3463-R | ATGATCCGCCTGCATCCGCT  CTGGTTGTCCAGCTGCTCGC | 216-bp DNA fragment spans nucleotides 34 to 249 bp of the *XC_3463*, used for RT-PCR. |
| 3575-F  3575-R | CCGCGTTGCAGGTCAAGGAA  ATGCCCAGCTTGCGCAGGTA | 217-bp DNA fragment spans nucleotides 89 to 305 bp of the *XC_3575*, used for RT-PCR. |
| 3591-F  3591-R | TGGTGATTGGCGTTGCCAGC  GATGCGTCCGTTGCTGCCAA | 179-bp DNA fragment spans nucleotides 56 to 234 bp of the *XC_3591*, used for RT-PCR. |
| 3754-F  3754-R | AGCAGTTTGGCGGACCGCAA  CCATGCCTTCGGAGAGCACC | 195-bp DNA fragment spans nucleotides 80 to 274 bp of the *XC_3754*, used for RT-PCR. |
| 16SF  16SR | GCCTAACACATGCAAGTCGAACGGC  AATATTCCCCACTGCTGCCTCCCG | 325-bp DNA fragment of the 16S rDNA sequence, used for RT-PCR. |
| 1355-F  1355-R | ATCAAGGAAGCCCTGAGCAAGAC  GTTGACCGAGGTGATCTTCAGCA | 181-bp DNA fragment spans nucleotides 114 to 294 bp of the *XC_1355* (*hlp*), used for qRT-PCR. |
| 1184-F  1184-R | CGAATCATATTGATCGAGGA  ATCAGCTCAAGTCCCTCTTC | 116-bp DNA fragment spans nucleotides 7 to 122 bp of the *XC_1184*, used for qRT-PCR. |
| 1005-F  1005-R | GGCGCTGGCGCAAAAGAAAG  GCGGCAGTTCACCATTGGAT | 107-bp DNA fragment spans nucleotides 129 to 235 bp of the *XC_1005*, used for qRT-PCR. |
| 3590-F  3590-R | GTACGGTTGTGGGCGTCGTT  TGTAAATGTCTGCCGCCGCC | 150-bp DNA fragment spans nucleotides 104 to 253 bp of the *XC_3590*, used for qRT-PCR. |
| 0247-F  0247-R | GATCGAGATCACCGGCCCTA  TAGGCGCGGTGGAATCTTCG | 101-bp DNA fragment spans nucleotides 297 to 397 bp of the *XC_0247*, used for qRT-PCR. |
| 1002-F  1002-R | ACTGCACGCACGCAAGTGGA  TCCAAGCGGTGCATCGTCGA | 102-bp DNA fragment spans nucleotides 100 to 201 bp of the *XC_1002*, used for qRT-PCR. |
| 1027-F  1027-R | GCGACCATGATCAAAGCCGG  GGCGGTGCTGTCTTCATCAC | 150-bp DNA fragment spans nucleotides 100 to 249 bp of the *XC_1027*, used for qRT-PCR. |
| 1104-F  1104-R | ACGATGCCTGACCCCTGGTT  TCGAAGTCGGCAGTACCACG | 110-bp DNA fragment spans nucleotides 13 to 122 bp of the *XC_1104*, used for qRT-PCR. |
| 3458-F  3458-R | AAGTCCACCTTGCTCAAGGC  ACTCCTGGAACACCACCACC | 118-bp DNA fragment spans nucleotides 127 to 244 bp of the *XC_3458*, used for qRT-PCR. |
| 1432-F  1432-R | GTCACCCTGACCACCCAGTT  ATCTGGTACAGGGTCTGCCC | 140-bp DNA fragment spans nucleotides 145 to 284 bp of the *XC_1432*, used for qRT-PCR. |

§The underlined sequences indicate the restriction sites for *Bam*HI, *Eco*RI, *Hin*dIII, *Pst*I and *Xba*I, respectively.
